# A tetrameric SpoVA2 membrane complex is required for DPA transport into *Bacillus anthracis* spores

**DOI:** 10.1128/mbio.03777-25

**Published:** 2026-02-26

**Authors:** Yuanchen Yu, Fernando H. Ramírez-Guadiana, Yongqiang Gao, David Z. Rudner

**Affiliations:** 1 Department of Microbiology, Harvard Medical School 1811 , Boston, Massachusetts, USA; Indiana University Bloomington, Bloomington, Indiana, USA

**Keywords:** spore resistance, spore germination, dipicolinic acid, YhcN, YlaJ

## Abstract

The small molecule dipicolinic acid (DPA) plays a critical role in bacterial spore resistance during dormancy and in the exit from quiescence during germination. In *Bacillus subtilis*, the *spoVA* locus is required for DPA import into the developing spore and has been implicated in its release during germination. The SpoVAC (C) and SpoVAEb (Eb) proteins form a membrane complex through which DPA transits, and the SpoVAD (D) protein binds the cytoplasmic face of the complex, resembling a plug. The human pathogen *Bacillus anthracis* and other members of the *Bacillus cereus* group have two *spoVA* loci. The *spoVA1* operon resembles *B. subtilis spoVA*, while the *spoVA2* locus is more distantly related and only shares the three core genes *C2*, *D2*, and *Eb2*. Here, we show that *spoVA2* is critical for DPA import and spore heat resistance, while either locus is sufficient for DPA export during germination. We report that a fourth protein (called NJ2) encoded in the *spoVA2* locus is part of the C2/D2/Eb2 complex and is predicted to bind the extracytoplasmic face of the membrane complex, resembling a cap. We show that NJ2 is important for DPA import during *B. anthracis* sporulation and essential in *B. subtilis* engineered to express *spoVA2* in place of its native *spoVA* locus. Finally, we identified an Eb2 mutant that bypasses the requirement for NJ2 in DPA import and show that the mutation impairs DPA export, providing additional support for a direct role of SpoVA complexes in the release of DPA during germination.

**IMPORTANCE:**

Spore resistance and exit from dormancy during germination are central to the transmission and pathogenesis of endospore-forming pathogens like *Bacillus anthracis*. Our understanding of the molecular mechanisms underlying these processes has principally been informed by studies in the non-pathogenic model *Bacillus subtilis*. Here, we identify and characterize a membrane complex in *B. anthracis* that is critical for spore resistance and spore germination that is absent from *B. subtilis*. We show that this complex is required for the accumulation of dipicolinic acid in the spore core during sporulation and functions in its release from the core during germination. A deeper understanding of the molecular mechanisms of *B. anthracis* sporulation and germination will facilitate the development of strategies for more effective disease prevention and treatment.

## INTRODUCTION

Spores are the primary infectious form of the human pathogen *Bacillus anthracis*, the etiological agent of anthrax (1). Infection typically begins when spores enter the host through skin abrasions, ingestion of contaminated food or water, or inhalation of airborne particles (1–3). Upon entry, spores are recognized and engulfed by host macrophages. The spores germinate within the macrophage, eventually escaping into surrounding tissues as vegetative bacilli. The transition from a dormant spore to an actively replicating cell marks the onset of anthrax pathogenesis and can lead to localized cutaneous or systemic infections, including gastrointestinal and inhalational anthrax. Although anthrax is widely recognized due to its potential use in bioterrorism and historical weaponization, the majority of human cases are associated with occupational or incidental contact with infected animals or their by-products, such as hides, wool, or meat (1, 2, 4). More recently, changing environmental conditions have begun to reshape the epidemiology of anthrax and other spore-forming pathogens. Climate change, deforestation, permafrost thawing, and increased encroachment into wildlife habitats due to agriculture and urbanization have all been implicated in the resurgence of seasonal anthrax outbreaks and the emergence of novel zoonotic pathogens of the *Bacillus cereus* group that includes *B. anthracis* (2, 5–9). A deeper understanding of the molecular mechanisms of *B. anthracis* spore resistance and spore germination will facilitate the development of new therapeutic interventions.

When nutrients become limiting, *B. anthracis* and other Bacilli and Clostridia differentiate into dormant spores that are resistant to desiccation, heat, irradiation, degradative enzymes, predation, and antibiotic treatment (10–13). Bacterial spores can remain dormant for decades, yet rapidly germinate and resume growth upon exposure to nutrients. Central to both spore resistance and the exit from dormancy is the small molecule dipicolinic acid (DPA) (12). DPA accumulates to 10%–15% of the dry weight of dormant spores during sporulation, where it displaces water, contributing to core dehydration and wet heat resistance. DPA levels scale with heat resistance. A reduction in DPA levels reduces spore heat resistance, and spores that lack DPA are fragile and easily lyse (14, 15). DPA is equally critical for the exit from dormancy (10, 12). Dormant spores germinate upon exposure to nutrient signals (called germinants) that are detected by receptors embedded in the spore inner membrane. Recent studies indicate that these receptors are ligand-gated ion channels and, in response to germinants, release ions from the spore core (16). Ion release, in turn, triggers the release of the large depot of DPA (16). As DPA transits the integument layers of the spore, it activates the cell wall-degrading enzyme CwlJ (17). CwlJ targets and degrades the specialized peptidoglycan that encases the spore, called the cortex, enabling core hydration and the resumption of macromolecular synthesis (10, 12). Thus, DPA is both critical for spore resistance during dormancy and for germination during the exit from dormancy.

In *Bacillus subtilis*, DPA is produced in the mother cell that nurtures the developing endospore at a late stage in sporulation. Two enzymes, SpoVFA and SpoVFB, catalyze the synthesis of DPA from dihydro-dipicolinic acid, an intermediate in the L-lysine and *meso*-diaminopimelate pathways (18). DPA is then transported into the developing spore. Transport across the outer spore membrane requires SpoVV, a member of the Gate family of concentrative nucleoside transporters (19). SpoVV is located in the outer spore membrane, and data suggest it transports DPA across this first membrane. DPA is then transported across the inner spore membrane in a manner that depends on the *spoVA* (*spo5A* or *5A*) operon (20, 21). Recent work indicates that three genes in this operon, *spoVAC* (*C*), *spoVAD* (*D*), and *spoVAEb* (*Eb*) are essential for DPA accumulation in the spore and likely function as the inner membrane DPA transporter (14). The membrane proteins C and Eb are predicted to form a membrane complex through which DPA is transported. D is a soluble protein that resides in the core and is predicted to interact with the C/Eb complex at the inner face of the membrane and occlude the membrane channel. Importantly, the 5A proteins are retained in the dormant spore, and evidence suggests they function in DPA export during germination (14, 22). Specifically, amino acid substitutions in C or D that are predicted to partially disrupt the interaction between the subunits in the complex not only impair DPA import during sporulation but also more rapidly initiate DPA release during germination (14). Thus, the 5A complex plays a central role in the acquisition of spore resistance during development and in the exit from dormancy during germination.

Interestingly, *Bacillus anthracis* and the other members of the *B. cereus sensu lato* group encode two *spoVA* loci, *spoVA1* (*5A1*) and *spoVA2* (*5A2*) (23). The *5A1* operon has the same seven genes as *B. subtilis 5A,* and the three core proteins (C1, D1, and Eb1) are, on average, 75% identical to their *5A*(*Bs*) counterparts. The *5A2* locus also has seven genes, but only *C2*, *D2*, and *Eb2* are similar to the genes in the *5A1* and *5A*(*Bs*) loci. C2, D2, and Eb2 are, on average, 53% identical to their *5A1* paralogs. Here, we investigated the roles of the *5A1* and *5A2* loci in *B. anthracis*. We show that *5A2* is both necessary and sufficient for DPA accumulation during sporulation, while either *5A1* or *5A2* supports DPA release during germination. We further show that a fourth gene in the *5A2* locus, *spoVANJ2* (*NJ2*), encodes a lipoprotein that resides in a complex with C2, D2, and Eb2 and is predicted to bind to the C2/Eb2 membrane channel on the extracytoplasmic face of the membrane. Sporulating cells lacking NJ2 accumulate lower levels of DPA and are heat sensitive. Using this heat sensitivity, we screened for spontaneous mutations that restore heat resistance and identified a point mutation in *Eb2*, further implicating NJ2 in DPA import. Finally, we show that the Eb2 mutant bypasses the need for NJ2 in DPA uptake and modestly slows DPA release during germination. The latter finding provides additional evidence in support of the model that 5A complexes are involved in DPA export during germination. Altogether, our data indicate that the tetrameric 5A2 complex plays a critical role in DPA import and spore resistance in *B. anthracis*.

## RESULTS

### A screen for sporulation genes in *Bacillus anthracis* identifies the *spoVA2* (*5A2*) locus

**Fig 1:**
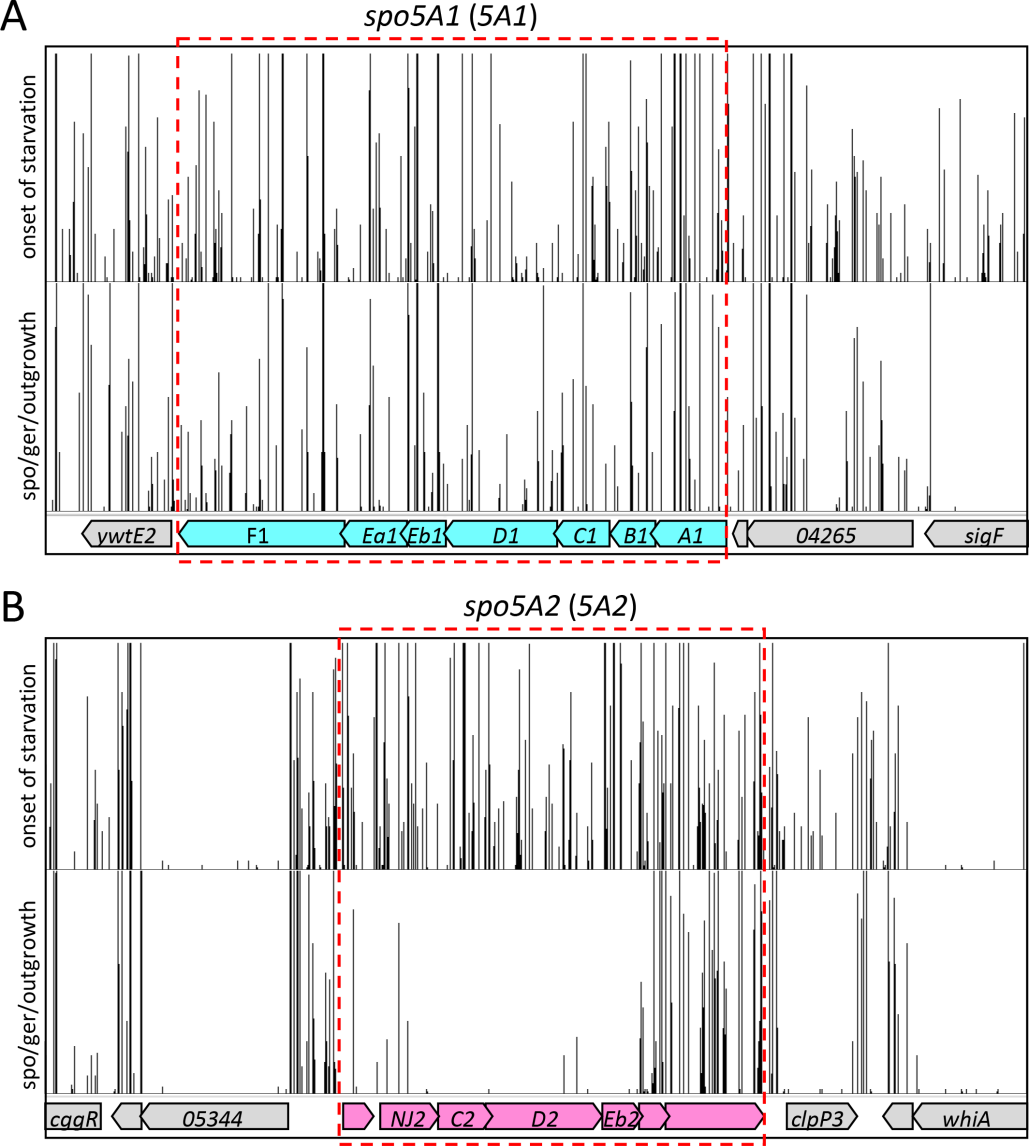
A transposon-sequencing screen identifies the *spo5A2* locus as important for sporulation. Transposon insertion profiles from *spo5A1* (**A**) and *spo5A2* (**B**) regions of the *B. anthracis* genome. A mariner-based transposon library was grown in PA medium. At the onset of starvation, a sample was collected. The remaining culture was sporulated for 7 h and then incubated at 65°C for 30 min to kill vegetative cells and sporulation-defective mutants. The surviving spores were plated on brain-heart infusion agar, and the resulting colonies were pooled. Transposon insertion sites and their abundance in the input and germinated and outgrown libraries were determined by deep sequencing and mapped to the reference genome. Each vertical line indicates a transposon insertion site. The height of the line reflects the number of sequencing reads at this position. The maximum height for each panel was set to 200 reads.

In a transposon-sequencing (Tn-seq) screen aimed at identifying sporulation genes in *Bacillus anthracis*, we discovered that genes in the *spoVA2* (*5A2*) operon, but not the *spoVA1* (*5A1*) operon, are important for sporulation (24). Figure 1 shows transposon insertion profiles for two regions of the *B. anthracis* genome that encompass the *5A1* and *5A2* loci. Transposon insertions in the *5A1* operon that were present at the onset of starvation were equally well represented after sporulation, exposure to heat, germination, and outgrowth (Fig. 1A). By contrast, insertions in several genes in the *5A2* locus were significantly underrepresented after the same treatment compared to the onset of starvation (Fig. 1B). Based on these findings, we initiated a thorough investigation of the role of the two loci in DPA import during sporulation and export during germination.

### The *5A2* locus is critical for DPA accumulation in dormant spores

**Fig 2:**
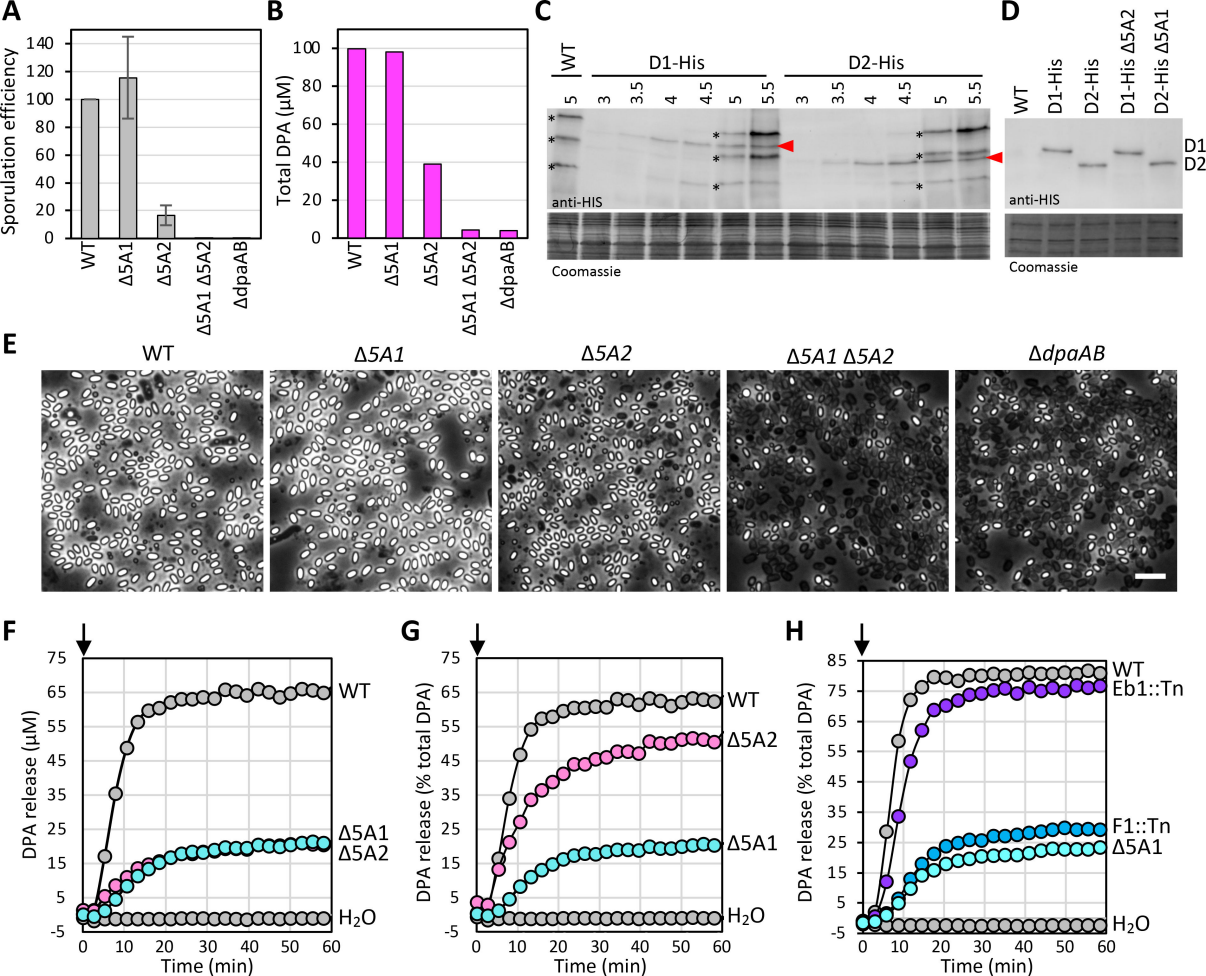
The *B. anthracis 5A2* locus is important for DPA accumulation in dormant spores. (**A**) Bar graph showing sporulation efficiencies of the indicated strains assessed by heat-resistant (65°C for 30 min) CFUs. Data presented are from three biological replicates (mean ± standard deviation). (**B**) Bar graph showing DPA levels in spores of the indicated strains. Spores were purified on a Histodenz step gradient, normalized, and boiled for 30 min to release DPA. The supernatant was mixed with TbCl_3_ and detected by fluorimetry. Data shown are averages of three technical replicates. A second biological replicate can be found in Fig. S1B. (**C**) Representative immunoblot of 5AD1-His and 5AD2-His during a sporulation time course in the indicated strains. Time (in hours) after the onset of sporulation is shown above the blot. Red carets indicate 5AD1-His and 5AD2-His bands. A lysate from wild-type (WT) was used to determine cross-reactive proteins (asterisks) recognized by the antibody. A portion of a Coomassie-stained gel with the same lysates was used to control for loading. (**D**) Representative immunoblot of 5AD1-His and 5AD2-His from spore lysates of the indicated strains. (**E**) Representative phase-contrast images of sporulated cells of the indicated strains. Scale bar, 5 μm. (**F–H**) Representative germination assays of the indicated strains. Purified spores were induced to germinate at time 0 (arrows) with 1 mM L-alanine and 1 mM inosine. The graph in panel **F** plots DPA release over time. The graphs in panels **G** and **H** plot the percentages of DPA that get released relative to the total DPA present in the spores for each strain. Total DPA and biological replicates of the strains in panel H can be found in Fig. S3. WT spores incubated in water were used as a control.

As a first step in our characterization of *5A1* and *5A2* operons, we generated strains with deletions of the entire *5A1* or *5A2* locus, as well as a double mutant, and tested them for sporulation efficiency. We sporulated the single and double mutants and the wild-type parental strain by nutrient exhaustion in PA medium for 30 h. The sporulated cultures were then incubated at 65°C for 30 min to kill vegetative cells and sporulating cells that failed to achieve heat resistance. The cultures were serially diluted and plated on brain-heart infusion (BHI) agar. After germination and outgrowth at 37°C, we enumerated the heat-resistant CFUs. Consistent with our Tn-seq results, the sporulation efficiency of the ∆*5A1* mutant was similar to that of the wild-type (Fig. 2A). By contrast, the sporulation efficiency of the ∆*5A2* strain was ~15% of wild-type, and the ∆*5A1* ∆*5A2* double mutant sporulated at <0.01%. We conclude that the *5A2* locus is important for proper spore formation, while the *5A1* locus is not.

Next, we investigated the requirement of each locus for DPA accumulation in dormant spores. The strains described above were sporulated on PA agar plates and spores were purified on a Histodenz step gradient. Equivalent amounts of phase-bright spores were boiled for 30 min to release their contents, and DPA was quantified from the supernatant using a TbCl_3_ fluorescence-based assay. Wild-type and the ∆*5A1* spores had similar levels of DPA (Fig. 2B). By contrast, the ∆*5A2* mutant spores had ~40% of the level of DPA found in wild-type. It was difficult to purify phase-bright spores that lacked both the *5A1* and *5A2* loci. Accordingly, the purified spores from the double mutant were a mixed population of phase-bright and phase-dark spores. These spores had nearly undetectable levels of DPA (Fig. 2B). For comparison, we measured the levels of DPA in spores lacking the DPA synthase genes *dpaA* and *dpaB* that encode homologs of *B. subtilis* SpoVFA and SpoVFB (18). The mixed population of phase-bright and phase-dark ∆*dpaAB* spores had similarly low levels of DPA (Fig. 2B). Collectively, these experiments indicate that the *5A2* locus is both necessary and sufficient for the accumulation of wild-type levels of DPA.

To investigate whether the ∆*5A2* mutant produces a mixed population of spores, some with wild-type levels of DPA and others with low levels of DPA (25), we took advantage of the reduction in heat resistance associated with lower levels of DPA (14, 15). Incubation of wild-type and ∆*5A1* spores at 75°C for 30 min yielded similar heat-resistant CFUs (Fig. S1). By contrast, virtually all ∆*5A2* mutant spores were killed by this treatment (Fig. S1). Although this result does not exclude the possibility that ∆*5A2* spores have heterogeneous levels of DPA, the loss of viability of virtually all mutant spores argues that ∆*5A2* spores have reduced levels of DPA compared to wild-type.

Finally, we used phase-contrast microscopy to visualize the sporulation cultures of wild-type and the mutants. As anticipated, the wild-type and ∆*5A1* sporulating cultures had an abundance of phase-bright spores, a hallmark of DPA accumulation in *B. subtilis* (Fig. 2E). However, unlike *B. subtilis*, the sporulating cultures of the ∆*5A2* single mutant and the ∆*5A1* ∆*5A2* and ∆*dpaAB* double mutants had a substantial number of phase-bright spores, given their sporulation efficiencies and levels of DPA. These findings indicate that the phase-bright appearance of *B. anthracis* spores is not a good proxy for DPA accumulation.

### Evidence that the 5A2 complex is more active than 5A1 in DPA import

One possible explanation for the more critical role of the *5A2* locus in DPA import is that the transport complex is produced at higher levels during spore formation than the 5A1 complex. To investigate this possibility, we compared the levels of the D subunits from each locus, using epitope-tagged variants. We appended hexa-histidine tags to the 3′ ends of the *D1* and *D2* genes at their native chromosomal locations. Importantly, both fusions were functional, based on sporulation efficiency, heat resistance at 75°C, and DPA accumulation (Fig. S2). We collected samples of the two strains over a sporulation time course and analyzed the levels of the D paralogs over time. The immunoblot in Fig. 2C shows that the D1-His and D2-His variants have similar intensities at each time point. Although we cannot exclude the possibility that the two D paralogs have different transfer efficiencies onto the PVDF membrane in our immunoblot, these data argue that both proteins are produced at similar levels throughout the differentiation process. Similarly, the band intensities of 5AD1-His and 5AD2-His in lysates from dormant spores harboring both complexes or only one were comparable (Fig. 2D). These findings suggest that 5A1 and 5A2 complexes are produced at similar levels and argue that the 5A2 complex is more active than the 5A1 complex in DPA transport into the developing spore.

### The 5A1 and 5A2 are both sufficient for DPA release during germination

To investigate whether 5A2 was more critical for spore germination than 5A1, we incubated purified spores from WT, ∆*5A1,* and ∆*5A2* with a germinant mixture of 1 mM L-alanine and 1 mM inosine and followed DPA release over time. For these assays, we did not heat-activate the spores because the ∆*5A2* mutant spores were heat-sensitive. As can be seen in Fig. 2F and G, wild-type spores released >60% of their DPA within 15 min of germinant exposure. Spores with only the *5A1* locus (∆*5A2*) released >50% of their DPA at a modestly reduced rate compared to wild-type. By contrast, spores with only *5A2* (∆*5A1*) released only 20% of their DPA and did so more slowly. We have previously shown that the last gene in the *B. subtilis 5A* operon, *spoVAF* (*F*), encodes an ion channel that, with its partner protein FigP, enhances the rate and extent of germination by releasing ions in response to ion release by the germinant receptors (26). To investigate whether the impaired DPA release from spores harboring only the *5A2* locus (∆*5A1*) was due to the absence of the C1/D1/Eb1 DPA transport complex or the F1/FigP ion channel, we performed additional germination assays using mutations of individual genes in the operon. As can be seen in Fig. 2H and Fig. S3, spore germination of *F1::Tn* and *figP::Tn* mutants resembled ∆*5A1* germination, while *D1::Tn* and *Eb1::Tn* mutants resembled wild-type. We conclude that the 5A2 transporter, like the 5A1 transporter, is proficient at DPA release during germination and that the F1 and FigP proteins function like their *B. subtilis* orthologs, amplifying the germinant response. Interestingly, the requirement for the putative F1/FigP channel in *B. anthracis* germination is even greater than the F/FigP channel in *B. subtilis* (26). Finally, these data indicate that, unlike *B. subtilis*, the *F1* gene is the most important gene for germination in the *5A1* locus in *B. anthracis*.

### The 5A2 transport complex contains C2, D2, Eb2, and NJ2

**Fig 3:**
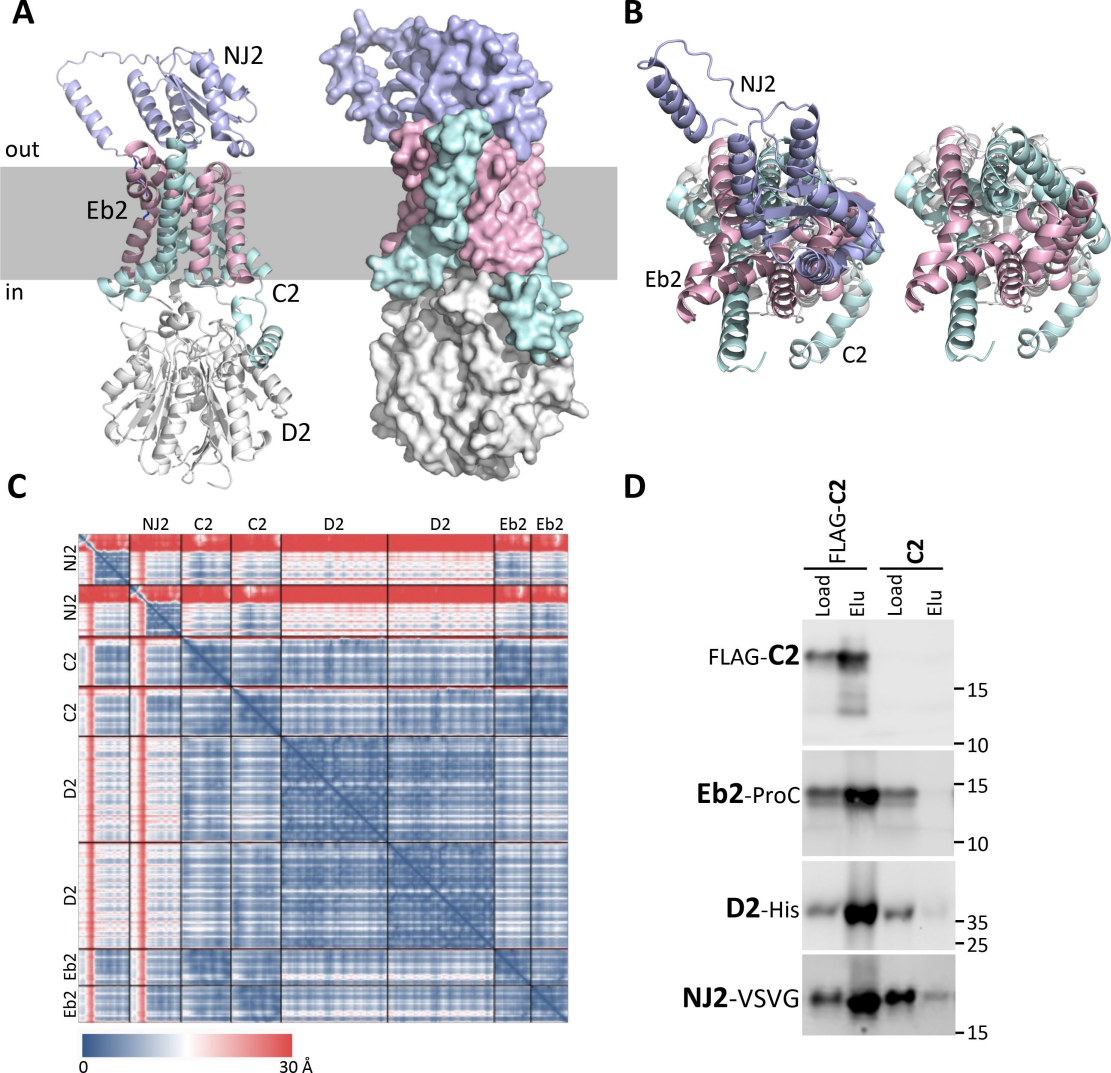
NJ2 resides in a complex with C2, D2, and Eb2. (**A**) Side view of the AlphaFold 3-predicted NJ2/C2/D2/Eb2 membrane complex. C2 (cyan) and Eb2 (pink) are predicted to form a membrane channel with interleaved transmembrane segments. D2 (white) is predicted to bind to the cytoplasmic face of the channel with contacts with the N-terminus of C2 in the cytosol. NJ2 (light purple) is predicted to bind the extracytoplasmic face of the C2/Eb2 channel, resembling a “cap.” The tetrameric complex is predicted to dimerize and is shown in Fig. S4. The tetramer is shown here for simplicity. (**B**) View of the complex with and without NJ2 from outside of the spore looking in. NJ2 is predicted to occlude the channel. (**C**) Predicted alignment error (pAE) in Å of all residues for the dimer-of-tetramer model. Low error (blue) corresponds to well-defined relative domain positions. The interface predicted template modeling (ipTM) score was 0.84. (**D**) Representative immunoblots of co-purification of the *B. cereus* NJ2/C2/D2/Eb2 complex. All four proteins were expressed in *E. coli,* and the detergent-solubilized membrane complex was purified on anti-M1 FLAG resin. C2 contains an N-terminal FLAG tag, D2 contains a C-terminal His tag, Eb2 contains a C-terminal Protein C (ProC) tag, and NJ2 contains a C-terminal VSVG epitope tag. A control strain expressing C2 lacking a FLAG tag was analyzed in parallel. For the D2-His and NJ2-VSVG immunoblots, 1/20 and 1/30, respectively, of the load was loaded compared to the FLAG-C2 and Eb2-ProC blots. Molecular weight markers (in kDa) are shown on the right of the blots.

We have previously shown that the C1, D1, and Eb1 proteins from *Bacillus cereus* reside in a membrane complex when expressed in *Escherichia coli* (14). Furthermore, the *B. subtilis* homologs are predicted by AlphaFold to form a high-confidence membrane complex that we validated by mutational analysis *in vivo* (14). Follow-up bioinformatic studies by Kilian and Bischofs found that the C/D/Eb trimer forms an even higher confidence dimer of trimers (27). We therefore investigated whether C2, D2, and Eb2 are predicted to interact. As anticipated based on the sequence similarities between homologs, AlphaFold 3 predicted a high-confidence dimer of trimers (Fig. 3A through C; Fig. S4). We also tested whether the other proteins encoded in the *5A2* locus interact with this core complex. Strikingly, AlphaFold 3 predicted a high-confidence interaction between the C2/D2/Eb2 membrane complex and the YhcN/YlaJ homolog encoded in the 5A2 locus, forming a dimer of tetramers (Fig. 3A through C; Fig. S4). The YhcN/YlaJ lipoprotein (28, 29), which we have renamed SpoVANJ2 (NJ2), is predicted to interact with the extracytoplasmic face of the C2/Eb2 membrane channel. As can be seen in Fig. 3A and B, NJ2 is predicted to occlude the channel, resembling a cap.

To experimentally determine whether NJ2 resides in a complex with C2/D2/Eb2, we co-expressed epitope-tagged variants of the four proteins in *E. coli*. For historical reasons, we used the *B. cereus* 5A2 proteins that are ~98% identical to the *B. anthracis* homologs (Fig. S5) (14). *E. coli* cells were induced to co-express FLAG-C2, D2-His, Eb2-ProteinC (ProC), and NJ2-VSVG or a control in which the C2 protein was untagged. Membrane preparations were generated from the *E. coli* lysates, and the membrane proteins were solubilized with the nonionic detergent n-dodecyl-β-D-maltoside (DDM). The DDM-solubilized lysates were then incubated with anti-FLAG resin and, after washing, were eluted with FLAG peptide. The eluates were then resolved by SDS-PAGE followed by immunoblotting. As can be seen in Fig. 3D, D2-His, Eb2-ProC, and NJ2-VSVG all co-purified with FLAG-C2. Importantly, these proteins were not found, or barely so, in the eluate of the purification from the untagged C2 strain (Fig. 3D). Collectively, these data strongly suggest that NJ2 resides in a membrane complex with C2, Eb2, and D2 and supports the AlphaFold-predicted model.

### NJ2 is important for DPA accumulation and heat resistance

**Fig 4:**
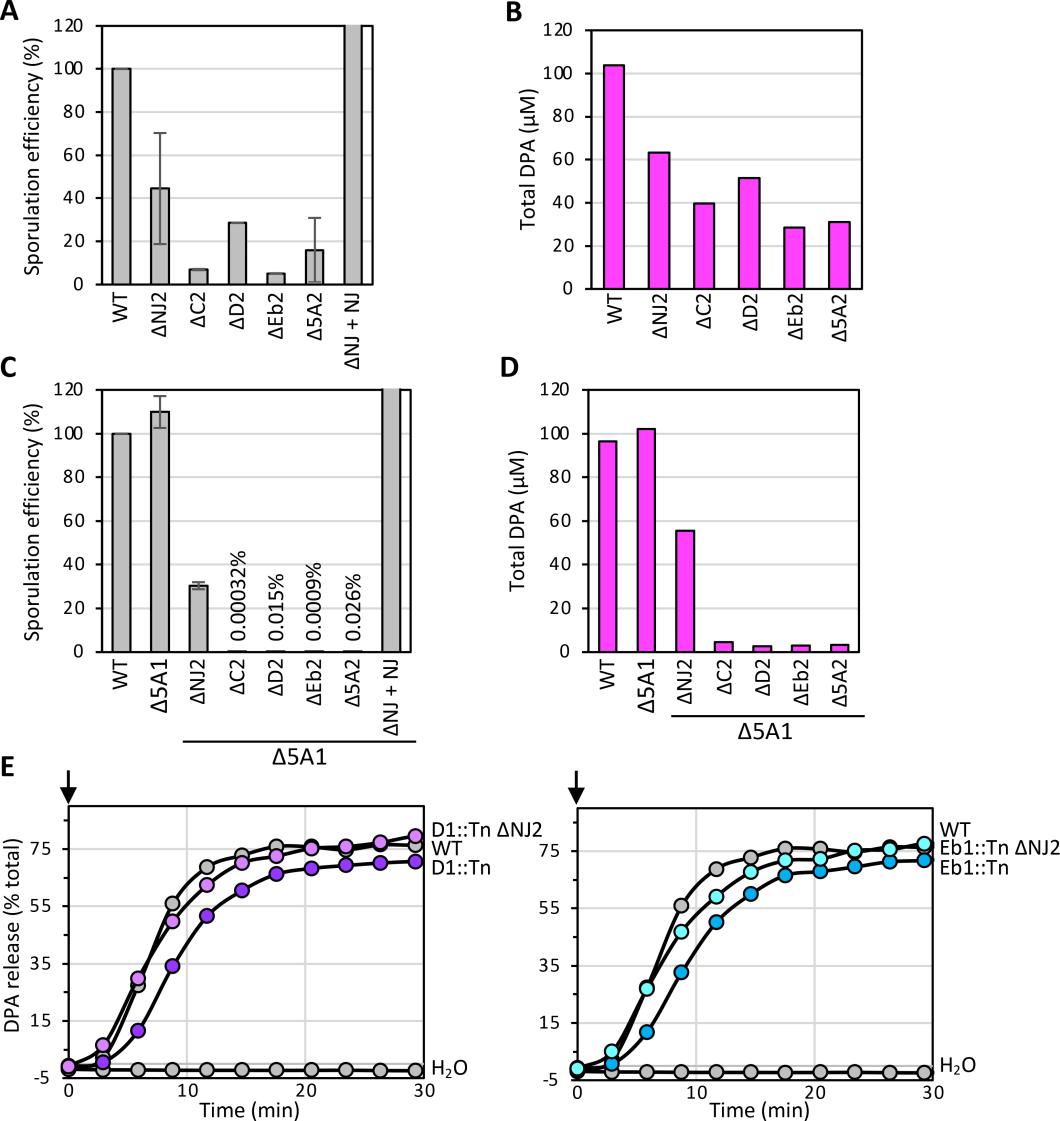
NJ2 is important for DPA import and impacts DPA export in *B. anthracis*. (**A**) Bar graph of sporulation efficiencies of the indicated strains, assessed by heat-resistant (65°C for 30 min) CFUs. Data are from two biological replicates (mean ± standard deviation). (**B**) Bar graph showing DPA levels in purified spores of the indicated strains. Spores were isolated on a Histodenz step gradient, normalized, and then boiled to release DPA. Data are averages of three technical replicates. (**C**) Bar graph of sporulation efficiencies of the indicated strains. Data are from three biological replicates (mean ± standard deviation). Sporulation efficiencies are shown above the indicated strains. (**D**) Bar graph showing DPA levels in spores of the indicated strains. Data are averages of three technical replicates. (**E**) Germination assays as monitored by DPA release. Purified spores of the indicated strains were induced to germinate by the addition of 1 mM L-alanine and 1 mM inosine (arrows). WT spores (gray circles) incubated with water were used as a control. DPA levels for panels E and 4F and biological replicates of panels B, D, and E can be found in Fig. S6.

To further explore the requirement of the *5A2* genes in DPA import, we generated strains with in-frame deletions of *NJ2*, *C2*, *D2*, or *Eb2*. We then analyzed sporulation efficiency of the individual mutants. Figure 4A shows that sporulating cells lacking *C2* or *Eb2* have sporulation efficiencies below 10%, similar to the ∆*5A2* mutant. Cells lacking *D2* sporulate at 25%, and cells lacking *NJ2* at ~40% efficiency. Importantly, expression of *NJ2 in trans* restored sporulation efficiency to wild-type levels, confirming that the defect is attributable to loss of NJ2. DPA accumulation in the mutant spores largely mirrored the sporulation efficiencies, with the ∆*NJ2* mutant accumulating ~60% of the level of DPA found in wild-type (Fig. 4B; Fig. S6C). Consistent with the reduced levels of DPA, all four single mutants were killed upon exposure to 75°C for 30 min (Fig. S6A).

Next, we combined the single gene deletions with a ∆*5A1* operon mutant. The double mutants with ∆*C2*, ∆*D2*, and ∆*Eb2* phenocopied the ∆*5A1* ∆*5A2* mutant with <0.1% sporulation efficiencies (Fig. 4C). Interestingly, the ∆*NJ2* ∆*5A1* mutant sporulated at ~30% efficiency, suggesting that the core complex of C2, D2, and Eb2 retains some DPA transport activity in the absence of NJ2 (Fig. 4C). Indeed, quantification of DPA accumulation in the in-frame deletion mutants with ∆*5A1* mirrored the sporulation efficiencies, with nearly undetectable levels of DPA in the double mutants with ∆*C2,* ∆*D2,* or ∆*Eb2* and ~50% DPA levels in the ∆*NJ2* ∆*5A1* mutant (Fig. 4D; Fig. S6D). These data indicate that the core complex of C2, D2, and Eb2 is capable of transporting DPA, albeit at reduced efficiency.

Finally, we analyzed DPA release from spores that had an intact 5A2 DPA transport complex and ones that lacked NJ2. Specifically, we compared *Eb1::Tn* and *D1::Tn* mutants with and without *NJ2*. As can be seen in Fig. 4E and S6E, wild-type and all four mutant strains released similar percentages of their DPA in response to nutrients. However, spores that lacked NJ2 released DPA more rapidly than those that contained this subunit (Fig. 4E; Fig. S6E). These findings suggest that the presence of the NJ2 “cap” on the 5A2 complex slows DPA release during germination.

Collectively, our findings argue that the NJ2 subunit of the DPA transport complex is not essential for DPA import but contributes to its efficiency. Furthermore, our analysis suggests that the NJ2 cap slows, albeit modestly, DPA release during germination.

### An Eb2 mutant restores DPA import activity to spores lacking NJ2

**Fig 5:**
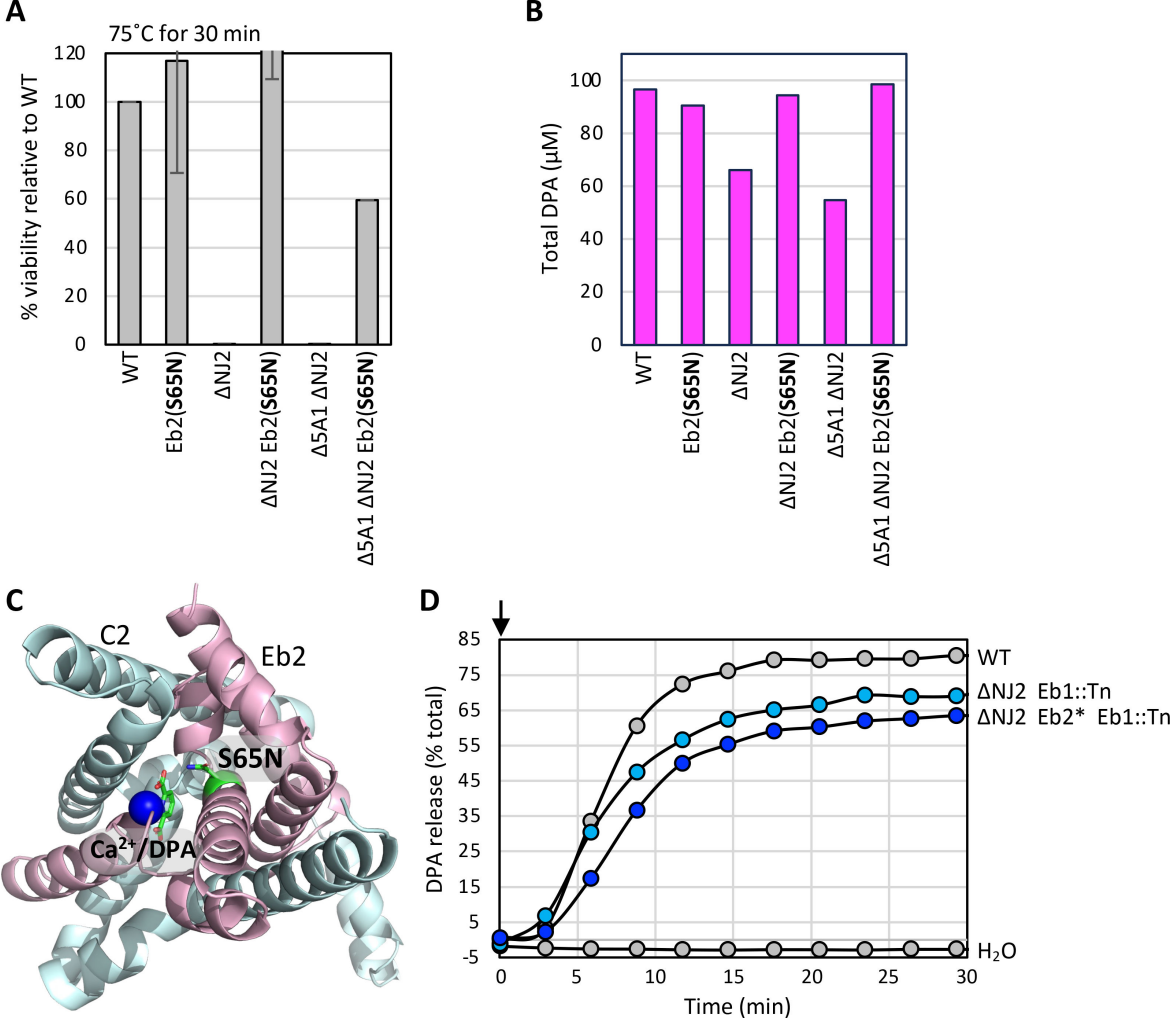
Eb2(S65N) bypasses the requirement for NJ2. (**A**) Bar graph of spore viability after exposure to 75°C for 30 min, as assayed by heat-resistant CFUs. Data represent two biological replicates (mean ± standard deviation). (**B**) Bar graph showing DPA levels in spores of the indicated strains. Spores were isolated on a Histodenz step gradient, normalized, and then boiled to release DPA. Data are averages of three technical replicates. (**C**) Predicted structure of the C2-Eb2(S65N) membrane complex with Ca^2+^/DPA, modeled using Chai-1. DPA is colored green, Ca^2+^ in blue, and S65N in green. (**D**) Spore germination in the indicated strains in response to 1 mM L-alanine and 1 mM inosine, as assayed by DPA release. Germinants were added at time 0 (arrow), and DPA release was monitored over time. The Eb2(S65N) (Eb2*) mutation modestly but reproducibly delayed DPA release. WT spores were incubated with water as a control. Biological replicates of panels B and D can be found in Fig. S5.

To provide more insight into NJ2’s role in DPA transport, we performed a genetic screen based on the heat sensitivity of ∆*NJ2* spores (Fig. S6A). We enriched for mutants that could survive 75°C for 30 min over successive rounds of sporulation, heat treatment, and reinoculation into sporulation medium. Fourteen independent cultures were subjected to five rounds of enrichment, and all 14 achieved high-level heat resistance. One colony from each culture was re-tested for its ability to produce heat-resistant spores. We then mapped the mutations from these 14 independent suppressor strains by whole-genome sequencing. Eleven strains had a frame-shift mutation in the *gerE* gene that increased or decreased the number of dinucleotide repeats at position 41 of the open reading frame (Fig. S7A). Identical loss-of-function mutations in *gerE* have been previously reported to confer heat resistance in *Bacillus cereus* (30). The other three independently isolated suppressors had an identical mutation in the *Eb2* gene resulting in a substitution of a conserved serine at position 65 to asparagine (S65N) (Fig. 5A; Fig. S7). In the AlphaFold-predicted model, S65 is present in the lumen of the C2/Eb2 channel near the opening that faces the intermembrane space (Fig. 5C). Intriguingly, Chai-1 (31) predicts that Ca^2+^/DPA is present in the C2/Eb2 channel in close proximity to S65 (Fig. 5C). A substitution for asparagine at this position could help stabilize DPA through an interaction between the amino group of the carboxamide and one of the carboxyl groups of DPA. We rebuilt this mutation by allelic exchange in the wild-type *5A2* locus and in the *5A2* locus lacking *NJ2*. The Eb2(S65N) mutant strain sporulated at wild-type levels, produced heat-resistant spores, and accumulated wild-type levels of DPA (Fig. 5A and B). The same was true for the strain lacking NJ2. Thus, an amino substitution within the predicted lumen of the C2/Eb2 channel improves DPA import in a strain lacking NJ2.

Interestingly, we discovered that purified ∆*NJ2* spores lacking a functional 5A1 transporter that harbor the Eb2(S65N) suppressor (called Eb2*) were modestly, but reproducibly, delayed in DPA release (Fig. 5D; Fig. S7D). Thus, improving DPA import during spore formation reduces the efficiency of export during germination. These findings define the first mutation in a *5A* gene that is specifically impaired, albeit modestly, in DPA export and provide additional support for the model that 5A complexes function to release DPA during germination.

### NJ2 is essential for DPA accumulation in *B. subtilis* spores that depend on the *5A2* operon

**Fig 6:**
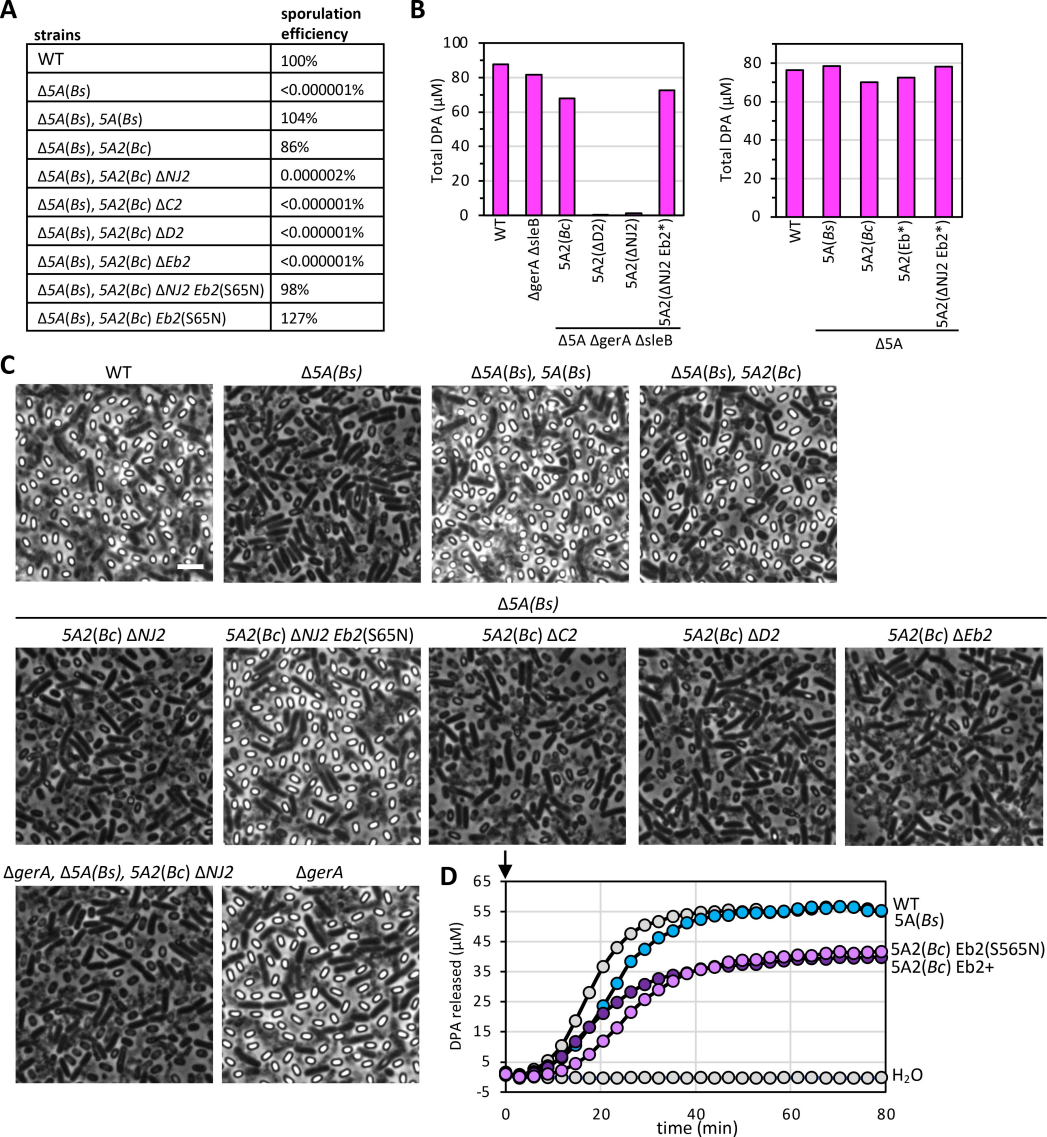
*NJ2* is required for DPA accumulation and spore heat resistance in *B. subtilis*. (**A**) Table of sporulation efficiencies of the indicated *B. subtilis* strains as assayed by heat-resistant (80°C for 20 min) CFUs. Data represent two biological replicates (mean ± standard deviation). The 5A2(*Bc*) locus from *B. cereus* complements a deletion of the native 5A(*Bs*) operon. (**B**) Bar graphs showing DPA levels in spores of the indicated strains. Eb2(S65N) (Eb2*) restores WT DPA levels. Data are averages of three technical replicates. Spores in the left panel were purified by incubation with lysozyme followed by SDS. Spores in the right panel were purified on a Histodenz step gradient. (**C**) Representative phase-contrast images of sporulated cultures of the indicated strains (scale bar, 5 μm). (**D**) Spore germination in response to L-alanine as assayed by release of DPA in the indicated strains. Spores were induced to germinate with 1 mM L-alanine at time zero (arrow), and DPA release was monitored over time. WT spores (gray circles) incubated with water were used as a control. The Eb2(S65N) spores released DPA more slowly than Eb2+. See Fig. S8 for two biological replicates of panel D.

We have previously shown that expression of the *B. cereus 5A1* or *5A2* locus in *B. subtilis,* fused to the *B. subtilis sspB* promoter, can compensate for the absence of the native *5A(Bs*) operon (16) (Fig. 6A). To investigate whether NJ2 is required for DPA import or export in *B. subtilis*, we generated in-frame deletions of *NJ2*, *C2*, *D2*, and *Eb2* in the *B. subtilis* complementation strain and tested the mutants for sporulation efficiency. Sporulating cells lacking any of these *B. cereus* genes, including *NJ2*, were unable to form dormant spores in the absence of the native *5A* locus (Fig. 6A). In all cases, the vast majority of spores in the sporulated cultures were phase-dark, a hallmark of a failure to accumulate DPA and inappropriate activation of SleB (14, 32) (Fig. 6B and C). One possible explanation for the strong sporulation defect in the ∆*NJ2* mutant in *B. subtilis* was that sporulating *B. subtilis* cells activate the GerA germinant receptor when DPA levels fail to reach a critical threshold in a timely manner (14, 32). However, and importantly, strains lacking *gerA* did not suppress the sporulation defect or the phase-dark appearance of the spores of ∆*NJ2* or any of the single mutants (Fig. 6C; Fig. S8A and B). Thus, these findings indicate NJ2 is essential for DPA import in *B. subtilis*.

Importantly, we found that the sporulation defect and failure to accumulate DPA in the ∆*NJ2* mutant was fully suppressed by the *Eb2*(S65N) suppressor (Fig. 6A through C). These findings provide additional support for NJ2’s role in DPA import. Finally, we tested whether spores with Eb2(S65N) are impaired in DPA release during germination, as we found in *B. anthracis*. Purified spores harboring the wild-type *B. cereus 5A2* locus or the *5A2* locus with the *Eb2*(S65N) allele were induced to germinate with 1 mM L-alanine, and DPA release was monitored over time. As can be seen in Fig. 6D and Fig. S8C, there was a modest but reproducible delay in DPA release when spores contained the Eb2(S65N) mutant.

## DISCUSSION

Here, we have shown that the *B. anthracis 5A2* operon is critical for DPA transport into dormant spores, while either *5A1* or *5A2* is sufficient for its release during germination. AlphaFold predictions and our experimental validation established that the 5A2 DPA transport complex is tetrameric and contains both a cytoplasmic “plug” (D2) and an extracytoplasmic “cap” (NJ2). Sporulating cells lacking NJ2 are impaired in DPA import, resulting in reduced DPA accumulation and decreased heat resistance. We hypothesize that DPA transport across the inner forespore membrane by the 5A2 complex involves an alternating access mechanism (33), in which continuous rounds of uncapping and re-capping, and unplugging and re-plugging of the C2/Eb2 membrane channel by NJ2 and D2, help prevent a fully open channel conformation that would allow DPA to flow down its concentration gradient. This and other models for how DPA is moved across the inner spore membrane await structural and functional studies in the future.

The *B. subtilis 5A* locus, as well as the *B. anthracis 5A1* and *Clostridioides difficile 5A* operons, all lack a gene encoding an NJ2 homolog, suggesting that these DPA transporters function without a cap. Interestingly, *B. subtilis* encodes four NJ2 paralogs, YhcN, YlaJ, YutC, and CoxA, that are all expressed during sporulation in the forespore compartment (34, 35). However, these paralogs are not predicted to interact with the C/D/Eb transport complex (Fig. S9). Furthermore, deletions of these genes do not impact DPA accumulation (28, 29). In addition, we have previously shown that expression of the *C. difficile 5A* locus in a *B. subtilis* strain lacking its native *5A* operon is sufficient for DPA transport (16), further arguing that a cap is not required for 5A transport activity. In these cases, we hypothesize that conformational changes in the C/Eb membrane channel act with the D plug to prevent a fully open conformation.

It is noteworthy that in *B. anthracis* and the other *B. cereus sensu lato* family members, the gene encoding the cortex lytic enzyme SleB, which degrades the specialized cell wall during germination, is present in an operon with an NJ2 homolog (called *ylaJ*) and its putative inhibitor *ypeB* (29). Recent work in *B. cereus* indicates that spores lacking *ylaJ* are impaired in germination in a manner that depends on SleB (36). Furthermore, AlphaFold predicts that YlaJ resides in a complex with SleB and YpeB (36). These findings have led to the hypothesis that this lipoprotein contributes to the stability or activation of SleB. Thus, distinct NJ2 paralogs function with the 5A2 DPA transport complex and the cortex lytic enzyme SleB in the *B. cereus sensu lato* group. By contrast, *B. subtilis* contains four NJ2 homologs (YhcN, YlaJ, YutC, CoxA), none of which is encoded in the *5A* or *sleB-ypeB* operons. Furthermore, the *B. subtilis* homologs are not directly involved in either DPA transport or SleB activity (28, 29). The specialization of the NJ2 homologs in the *B. cereus* group, or the loss of this specialization in *B. subtilis*, represents a noteworthy difference between sporulation and germination in these endospore formers. Notably, *B. anthracis* also contains YutC and CoxA homologs, but their functions have not been investigated.

This study and our previous work on DPA transport (16) highlight both the strengths and limitations of *trans*-species complementation experiments. In previous studies, we showed that expression of the *5A1* or *5A2* operons from *B. cereus,* or the *5A* locus from *C. difficile,* in a *B. subtilis* strain lacking its native *5A* operon is sufficient for DPA import into spores (16). The *5A1* operon from *B. anthracis* and *B. cereus* are ~98% identical, and it is likely that the *B. anthracis 5A1* locus would also function in *B. subtilis*. Yet, the data presented here indicate that *B. anthracis* cells that lack *5A2* and rely on *5A1* for DPA import fail to accumulate wild-type levels of this metabolite in dormant spores. In this case, we suspect that the discrepancy can be explained by the strong promoter (P*sspB*) that we fused to the *5A1* operon to express it in *B. subtilis*. However, the data presented here reveal a second example where the phenotype in the pathogen is different from the *trans*-complemented model. In *B. anthracis*, the absence of NJ2 impairs, but does not abolish, DPA accumulation. By contrast, we found that NJ2 is absolutely essential for the function of 5A2 transport complex in *B. subtilis*. In its absence, the spores fail to accumulate DPA, and the mutant phenotype is virtually indistinguishable from deletions of *C2*, *D2*, or *Eb2*. We cannot account for the different requirements for NJ2 in the two systems, but these findings emphasize the importance of investigating gene function in the native organism.

Finally, our study provides an additional argument that 5A complexes function in DPA release during germination. Since 5A complexes are essential for DPA transport into spores, it has been challenging to establish whether they also function in DPA release. We have previously shown that amino acid substitutions in the *B. subtilis* C and D proteins that are predicted to weaken their interaction are impaired in DPA import. Purification of the mutant spores that achieved wild-type levels of DPA revealed that these spores initiate DPA release more rapidly in response to germinants (14). The germination phenotypes were modest but provided support for the idea that the 5A complex is directly involved in DPA release during germination. Here, we show that a point mutation in *Eb2* that bypasses the requirement for NJ2 accumulates wild-type levels of DPA but is impaired, albeit modestly, in DPA release. This represents the first example of a mutant in a 5A complex that is specifically impaired in DPA export. Thus, these findings add to the growing body of genetic evidence that 5A complexes function in DPA release during germination.

### Conclusion

We have shown that the *5A2* locus encodes a DPA transport complex in which the C2 and Eb2 subunits are predicted to form a membrane channel, and D2 and NJ2 bind to the cytoplasmic and extracytoplasmic faces of the membrane complex. Our data add to the growing body of evidence that these complexes import DPA into the developing spore during sporulation and release it during germination. The challenges for the future include testing this model *in vitro* in a reconstituted system and establishing how the 5A complexes are activated in response to ion release by the germinant receptors. We note that AlphaFold predicts that 5A2 tetramers, like the 5A1 (and 5A) trimers, dimerize (27), forming adjacent transport complexes. The function of dimerization is currently unknown, but it could relate to sensing ion release or to mechano-sensation that triggers channel opening during germination.

## MATERIALS AND METHODS

### Bacterial strains and media

All *Bacillus anthracis* strains in this study were derived from BaR1, the plasmid-free non-pathogenic Sterne derivative *B. anthracis* 9131 (37). *B. anthracis* cells were cultured in BHI broth or agar. When required, BHI medium was supplemented with chloramphenicol (5 µg/mL), erythromycin (5 µg/mL), kanamycin (25 µg/mL), or spectinomycin (300 µg/mL). Sporulation of *B. anthracis* was induced at 37°C by nutrient exhaustion in phage assay (PA) medium (38). All *Bacillus subtilis* strains in this study were derived from *B. subtilis* 168 (39). *B. subtilis* strains were grown in Luria-Bertani (LB) broth or agar. When required, LB medium was supplemented with chloramphenicol (5 µg/mL), erythromycin (1 µg/mL) plus lincomycin (25 µg/mL), or spectinomycin (100 µg/mL). Sporulation of *B. subtilis* was induced at 37°C by nutrient exhaustion in supplemented Difco Sporulation Medium (DSM) (40). Sporulation efficiency was determined in 30 h cultures as the total number of heat-resistant (*B. anthracis:* 65°C or, where indicated, 75°C for 30 min, and *B. subtilis*: 80°C for 20 min) CFUs compared to wild-type heat-resistant CFUs. *B. anthracis* transposon-insertion mutants were from the ordered Tn library generated by Ramirez-Guadiana et al. (24). *B. subtilis* insertion-deletion mutants were from the *Bacillus* knock-out (BKE) collection (41) or were generated by direct transformation of plasmids into *B. subtilis*. Tables of strains (Table S1), plasmids (Table S2), and oligonucleotide primers (Table S3) and a description of plasmid constructions can be found in Supplemental methods.

### CP51-mediated phage transduction in *B. anthracis*

A temperature-sensitive mutant of the generalized transducing phage CP51 was used to transduce antibiotic-marked alleles (38) into *B. anthracis* strains. Briefly, the donor strain was grown in BHI supplemented with 0.5% glycerol, then mixed with phage CP51 and PA soft agar. The mixture was poured onto nutrient broth/yeast extract/glycerol agar plates and incubated overnight at 30°C. The transducing lysate was prepared by collecting the soft agar and resuspending it in BHI supplemented with 0.5% glycerol, 10% dimethyl sulfoxide, and 20 mM MgSO_4_, followed by filtration through a 0.45 μm filter. The recipient strain was grown in BHI supplemented with 0.5% glycerol, mixed with the transducing lysate, and incubated with constant agitation at 37°C for 45 min. The mixture was then plated on BHI agar plates supplemented with the appropriate antibiotic and incubated overnight at 37°C. Transductants were confirmed by PCR.

### Electroporation

Transposon and allelic exchange plasmids, prepared from the *E. coli* strain SC110 (*dam*-, *dcm*-), were transformed into *B. anthracis* by electroporation. Electrocompetent cells were prepared by growing *B. anthracis* in BHI with 0.1% glucose (BHIG) and 0.005 µg/mL tunicamycin. Low concentrations of tunicamycin increase transformation efficiency ~30-fold, likely due to the partial inhibition of TagO, the committing enzyme for the synthesis of secondary cell wall polysaccharides (42, 43). Cells were harvested at an OD_600_ of 0.3, chilled on ice, and washed four times in cold electroporation buffer (0.5 M sucrose, 10% glycerol). One hundred microliters of cells (concentrated 1,000-fold) was mixed with ~2 µg plasmid DNA and transferred to a cold 1 mm electroporation cuvette. The electroporator settings were 25 µF, 200 Ω, 1.85 kV. Immediately after electroporation, cells were resuspended in 1 mL recovery medium (BHIG supplemented with 20 mM MgCl_2_ and 2 mM CaCl_2_) and incubated with constant agitation for 2 h at 30°C. The culture was plated on BHI agar supplemented with the appropriate antibiotic and incubated overnight at 30°C.

### Allelic exchange

Deletions, His_6_-fusions, and integration of transgenes at neutral chromosomal loci were generated by allelic exchange using pMiniMAD3 (a gift from Daniel Kearns) or pFR50, both derived from pMAD (44). The pMiniMAD3 vector carries a thermosensitive origin of replication and a spectinomycin resistance cassette for *Bacilli*. The pFR50 vector carries the same thermosensitive origin, an erythromycin resistance cassette for *Bacilli*, and mCherry fused to the *B. subtilis veg* promoter. DNA fragments of 1 kb flanking the deletion or insertion site were subcloned into these vectors, with or without a second antibiotic resistance cassette between them. The plasmids were prepared in *E. coli* strain SC110 and electroporated into *B. anthracis* followed by selection on BHI agar supplemented with spectinomycin or erythromycin. A colony was then grown in 5 mL BHI-spec or BHI-erm at 30°C. After 10 h of growth, the culture was serially diluted and plated on BHI-spec or BHI-erm plates, and incubated overnight at 42°C to select for cells with a single crossover integration (loop-in). A single colony was then grown without selection in 5 mL BHI at 30°C for 24 h. The culture was serially diluted and plated on BHI agar or BHI supplemented with the second antibiotic. Plates were incubated overnight at 37°C. Colonies were screened for loop-out by their sensitivity to spectinomycin or erythromycin. Allelic exchange was confirmed by PCR.

### Transposon library construction and transposon-insertion sequencing

Tn-seq was performed as described previously (45–47) with modifications described below. The *B. anthracis* transposon library was generated using the transposon delivery plasmid pFR38. This *E. coli-Bacillus* shuttle vector contains a temperature-sensitive replicon for *Bacilli*, an erythromycin resistance cassette, the mariner-*Himar1* transposase C9, and a spectinomycin resistance cassette flanked by inverted repeats recognized by the transposase. One of the two inverted repeats contains an MmeI endonuclease restriction site. pFR38 was introduced into the *B. anthracis* wild-type strain BaR1 via electroporation. Transformants were selected at 30°C on BHI agar supplemented with erythromycin. Ten test tubes with 4 mL of BHI supplemented with spectinomycin were each inoculated with four transformants and grown at 22°C for 16 h. The cultures were pooled, and transposants were selected on BHI agar plates supplemented with spectinomycin at 42°C. Approximately 1,000,000 transposant colonies were pooled, diluted to an OD_600_ of 5, distributed into 500 µL aliquots, and frozen in 15% glycerol at −80°C.

A frozen aliquot (500 µL) of the Tn-library was thawed, washed twice in PA medium, and diluted into 50 mL PA medium to an OD_600_ of 0.05. The culture was grown at 37°C, and growth was monitored by OD_600_ over time. An aliquot was harvested at the onset of starvation (input), and the rest of the culture was sporulated for 7 h. Spores from the BaR1 strain are less adherent to culture tubes after 7 h of sporulation, and the maximum number of heat-resistant CFUs can be recovered. The sporulated sample was incubated at 65°C for 30 min to kill vegetative cells and sporulation-defective mutants and plated on BHI agar. Approximately 1,000,000 heat-resistant colonies were pooled (output). gDNA was isolated from the input and output samples and digested with MmeI, followed by barcoded adaptor ligation. The transposon-chromosome junctions were amplified in 18 PCR cycles using a universal primer that binds the adaptor and a primer that binds within the transposon. The PCR product was gel-purified and sequenced on the Illumina HiSeq platform using TruSeq reagents (Tufts University Core Facility [TUCF]). The reads were mapped to the *B. anthracis* BaR1 genome, tallied at each TA site, and genes in which reads were statistically underrepresented were identified using the Mann-Whitney U-test. Visual inspection of transposon insertion profiles was performed with the Sanger Artemis Genome Browser and Annotation Tool (48).

### Spore purification

Strains were grown in liquid sporulation medium (PA for *B. anthracis*; DSM for *B. subtilis*) at 37°C to an OD_600_ of 0.6 and then spread on PA or DSM agar plates and incubated for 96 h at 30°C. Spores were harvested and washed three times with ddH_2_O. The spore pellet was resuspended in 20% Histodenz (Sigma-Aldrich), and 350 µL of the suspension was layered on top of 1 mL of 50% Histodenz. The step gradient was subjected to centrifugation at 15,000 rpm for 30 min at 4°C. The spore pellet was collected and washed four times with ddH_2_O. Except where indicated, the pellet fraction contained >95% phase-bright spores as assayed by phase-contrast microscopy. Spores were imaged and assayed on the same day they were purified.

### DPA quantification

Purified spores were normalized to an OD_600_ of 1 in 350 µL of ddH_2_O and incubated for 30 min at 100°C to release DPA. After centrifugation (15,000 rpm for 5 min), 75 μL of the supernatant was added to a black flat-bottom 96-well plate, with 75 μL of 100 μM TbCl_3_. The fluorescence signal was measured at 545 nm with excitation set at 272 nm using an Infinite M Plex plate reader (Tecan). Each sample was analyzed in technical triplicates and compared with a standard curve to determine the DPA concentration.

### DPA release in response to germinants

Purified spores were normalized to OD_600_ of 1 in ddH_2_O. *B. subtilis* spores were heat-activated for 30 min at 70°C, followed by incubation for 15 min on ice. Because *B. anthracis 5A2* mutant spores are heat-sensitive, *B. anthracis* spores were not heat-activated prior to the germination assay. Seventy-five microliters of spore suspension was transferred to a black, flat-bottom, 96-well plate. An equal volume of ddH_2_O or germinant (2 mM L-alanine + 2 mM inosine for *B. anthracis*; 2 mM L-alanine for *B. subtilis*) containing 100 µM TbCl_3_ was added to initiate germination. The fluorescence was monitored at 545 nm with excitation at 272 nm every 2 min for 3 h in an Infinite M Plex plate reader (Tecan). The 96-well plate was maintained at 30°C and agitated between measurements. All spore samples and conditions were tested in technical triplicate and compared with a standard curve to determine DPA release.

### Microscopy

Purified spores and sporulation cultures were concentrated by centrifugation and then immobilized on 1.5% agarose pads. Phase-contrast microscopy was performed using a Nikon Ti2-E inverted microscope equipped with a Plan Apo 100×/1.45 Oil Ph3 objective and Hamamatsu ORCA-Flash4.0 V3 Digital CMOS camera. The exposure times for phase-contrast were 200 ms. Image analysis and processing were performed using Fiji software.

### Enrichment screen for ∆NJ2 suppressors

Independent *B. anthracis* colonies were separately sporulated in 14 × 2 mL PA medium for 24 h at 37°C. The sporulated cultures were incubated at 75°C for 30 min (cycle 1), and then 500 µL were diluted into 2.5 mL fresh PA medium and incubated at 37°C for 24 h. After germination, outgrowth, and sporulation, the culture was again incubated at 75°C for 30 min (cycle 2), and then 500 µL were diluted into 2.5 mL fresh PA medium and incubated at 37°C. After five cycles, the cultures were serially diluted and plated on BHI agar plates. All 14 cultures achieved wild-type levels of heat resistance. A single colony from each culture was re-tested for heat resistance at 75°C, and the suppressor mutations were mapped by whole-genome sequencing.

### Immunoblot analysis

For sporulating cultures, 1 mL of the cultures was collected by centrifugation and the cell pellets were incubated with 50 µL of lysis buffer (20 mM Tris pH 7.5, 10 mM EDTA, 10 mM MgCl_2_, 1 mg/mL lysozyme, 10 μg/mL DNase I, 100 μg/mL RNase A, and 1 mM PMSF) for 10 min at 37°C followed by the addition of 50 µL of 2× sample buffer (4% SDS, 250 mM Tris pH 6.8, 20% glycerol, 10 mM EDTA, and Bromophenol blue) containing 10% β-mercaptoethanol. For Histodenz-purified spores, spores (OD_600_=20) were resuspended in 500 µL of 1× PBS supplemented with 1 mM PMSF and transferred to 2 mL tubes containing Lysing Matrix B (MP Biomedicals). The spores were lysed using a FastPrep (MP Biomedicals) with 6.5 m/s for 60 s, followed by incubation on ice. An equal volume of 2× sample buffer containing 10% β-mercaptoethanol was immediately added to the lysate. After centrifugation (5,000 rpm for 5 min), the supernatant was collected, and total protein was determined using a noninterfering protein assay (G-Biosciences). Protein concentrations were normalized, samples were heated at 95°C, and resolved by SDS-PAGE on 17.5% polyacrylamide gels and transferred to an Immobilon-P membrane (Millipore). A second gel containing the same samples was stained for 1 h with InstantBlue Coomassie (ISB1L) to control for loading. The membrane was blocked in 5% nonfat milk in 1× PBS with 0.5% Tween-20 (PBST) and probed with anti-His (1:4,000; GenScript) diluted in 3% BSA in PBST. The primary antibodies were detected with anti-mouse (1:10,000) secondary antibodies coupled to horseradish peroxidase (Bio-Rad) and detected using Western Lightning ECL reagent (PerkinElmer).

### Structural modeling

Structural modeling was performed using AlphaFold 3.0 (49) and Chai-1 (31). pAE plots were generated using https://thecodingbiologist.com/tools/pae.html.

### Co-purification of *B. cereus* FLAG-C2, NJ2-VSVG, D2-His, and Eb2-ProC

Plasmids pYG702 (*kan*) and pYG084 (*amp*) or pYY057 (*amp*) were co-transformed into *E. coli* BL21(DE3) containing pAM174 (*cat*) (50). The resulting expression strains were grown in 1 L Terrific broth supplemented with 0.4% glycerol, 0.1% glucose, 1 mM MgCl_2_, 100 μg/mL ampicillin, 25 μg/mL kanamycin, and 20 μg/mL chloramphenicol at 37°C with agitation until an OD_600_ of 0.2. The cultures were then transferred to a 20°C shaking incubator. When the OD_600_ reached 0.7, IPTG (0.5 mM final) and arabinose (0.1% final) were added. Eighteen hours after induction, cells were harvested by centrifugation at 8,000 × *g* for 15 min at 4°C. The cell pellet was resuspended in 45 mL of lysis buffer (50 mM HEPES at pH 7.6, 150 mM NaCl, 20 mM MgCl_2_, 0.5 mM DTT) with 5 U/L benzonase (Sigma E1014), and lysed by two passages through a cell disruptor (Constant Systems) at 25,000 psi. The lysate was subjected to ultracentrifugation at 35,000 rpm for 1 h at 4°C. The membrane pellet was homogenized in solubilization buffer (20 mM HEPES at pH 7.6, 150 mM NaCl, 20% glycerol, 1% n-dodecyl-β-D-maltopyranoside [DDM; Thermo Fisher]) and rotated for 1 h at 4°C. The mixture was subjected to ultracentrifugation at 35,000 rpm for 1 h at 4°C, and the supernatant was collected and supplemented with 2 mM CaCl_2_. The DDM-solubilized proteins were loaded on M1 α-Flag antibody resin (1 mL bed volume). Bound proteins were washed with 25 column volumes (CVs) of wash buffer (20 mM HEPES at pH 7.6, 150 mM NaCl, 20% glycerol, 2 mM CaCl_2_, 0.1% DDM), and then eluted from the column with five CVs of elution buffer (20 mM HEPES at pH 7.6, 150 mM NaCl, 0.1% DDM, 5 mM EDTA pH 8.0, 0.2 mg/mL FLAG peptide). The eluted proteins were resolved by SDS-PAGE on a 17.5% polyacrylamide gel and visualized by InstantBlue Coomassie staining (ISB1L) or immunoblot as described above. Prior to SDS-PAGE, samples were incubated at 95°C for 5 min when probing for D2-His and NJ2-VSVG, and 50°C for 15 min when probing for FLAG-C2 and Eb2-ProC. Primary antibodies were anti-His (1:2,000; GenScript), anti-VSVG (1:1,000; Sigma), anti-FLAG (1:5,000; Sigma), and anti-Protein C (1:1,000; Sigma).

## SUPPLEMENTAL MATERIAL

Supplemental Information mbio.03777-25-s0001.pdf Supplemental methods, figures, and tables.

ASM does not own the copyrights to Supplemental Material that may be linked to, or accessed through, an article. The authors have granted ASM a non-exclusive, world-wide license to publish the Supplemental Material files. Please contact the corresponding author directly for reuse.
