# Supplementary material for: A tetrameric SpoVA2 membrane complex is required for DPA transport into Bacillus anthracis spores
Source: mBio. 2026 Feb 26;17(4):e03777-25. doi: 10.1128/mbio.03777-25 (PMC13059800; doi:10.1128/mbio.03777-25)
Supplement: Supplemental Information — Supplemental methods, figures, and tables. [file mbio.03777-25-s0001.pdf]

SUPPORTING INFORMATION

A tetrameric SpoVA2 membrane complex is required for DPA transport into *Bacillus anthracis* spores

Yuanchen Yu, Fernando H. Ramírez-Guadiana, Yongqiang Gao, and David Z. Rudner

Department of Microbiology, Harvard Medical School
77 Avenue Louis Pasteur
Boston, MA 02115

Supporting information includes:

Supplemental Methods
Supplemental Figures (S1-S9)
Supplemental Tables (S1-S3)

SUPPLEMENTAL METHODS

Plasmid constructions

pFR38 [Himar1C9 IR-spec (*amp, erm*)] was constructed via a two-way ligation of a PstI-HindIII fragment containing a spectinomycin resistance cassette flanked by two inverted terminal repeats, one of which contained an MmeI site, into pMarA digested with the same enzymes.

pFR50 [pMiniMAD P_{veg}-*mCherry* (*amp*, *erm*)] was constructed via a two-way ligation of an EcoRI-BamHI fragment from pER75 containing the *veg* promoter from *B. subtilis* fused to *mCherry* and pMiniMAD digested with the same enzymes. pER75 [*sacA*::*Pveg-mCherry* (kan)] is a *B. subtilis* double crossover integration vector (Rudner Lab stock).

pBaR5 [pMiniMAD P_{veg}-*mCherry* 395/396::*kan* (*amp, erm*)] was assembled in two steps. First, two fragments of 1,019 bp spanning the intergenic region between the convergently transcribed genes *00395* and *00396* were PCR-amplified from *B. anthracis* BaR1 with oligonucleotide primer pairs oFR295+oFR296 and oFR297+oFR298. These PCR products correspond to upstream (BamHI-ApaI) and downstream (ApaI-SalI) flanking regions, respectively. The two fragments were ligated into pFR50 digested with BamHI and SalI. In the second step, a kanamycin resistance cassette, flanked by strong transcriptional terminators, was cut with ApaI and inserted into the intermediate construct digested with ApaI.

pYY31 [pMiniMAD Δ*spoVANJ2* (amp, spec)] was constructed in a 3-way ligation with two PCR products and pMiniMAD3 cut with SalI and EcoRI. The two PCR products were the regions upstream and downstream of *spoVANJ2*, amplified from BaR1 genomic DNA using oYY158+oYY159 and oYY160+oYY161. Ligation of these regions generated an in-frame deletion of *spoVANJ2*.

pYY32 [pMiniMAD Δ*spoVAC2* (*amp*, *spec*)] was constructed in a 3-way ligation with two PCR products and pMiniMAD3 cut with SalI and EcoRI. The two PCR products were the regions upstream and downstream of *spo5AC2,* amplified from BaR1 genomic DNA using oYY154+oYY155 and oYY156+oYY157. Ligation of these regions generated an in-frame deletion of *spoVAC2*.

pYY33 [pMiniMAD Δ*spoVAD2* (*amp*, *spec*)] was constructed in a 3-way ligation with two PCR products and pMiniMAD3 cut with SalI and EcoRI. The two PCR products were the regions upstream and downstream of *spo5AD2,* amplified from BaR1 genomic DNA using oYY147+oYY148 and oYY150+oYY153. Ligation of these regions generated an in-frame deletion of *spoVAD2*.

pYY35 [pMiniMAD Δ*spoVAEb2* (*amp*, *spec*)] was constructed in a 3-way ligation with two PCR products and pMiniMAD3 cut with SalI and EcoRI. The two PCR products were the regions upstream and downstream of *spo5AEb2,* amplified from BaR1 genomic DNA using oYY166+oYY167 and oYY168+oYY169. Ligation of these regions generated an in-frame deletion of *spoVAEb2*.

pYY36 [pMiniMAD Δ*spoVA1* (*amp*, *spec*)] was constructed in a 3-way ligation with two PCR products and pMiniMAD3 cut with SalI and EcoRI. The two PCR products were the regions upstream and

downstream of *spoVA1,* amplified from BaR1 genomic DNA using oYY172+oYY173 and oYY174+oYY175.

pYY37 [pMiniMAD Δ*spoVA2* (*amp*, *spec*)] was constructed in a 3-way ligation with two PCR products and pMiniMAD3 cut with SalI and EcoRI. The two PCR products were the regions upstream and downstream of *spoVA2,* amplified from BaR1 genomic DNA using oYY162+oYY163 and oYY170+oYY171.

pYY40 [pMiniMAD Δ*spoVA1*::cat (*amp*, *spec*)] was constructed in a 2-way isothermal assembly reaction with PCR a product and pYY36 cut with BamHI. The *cat* cassette was amplified from pWX465 using oYY190 and oYY191. pWX465 contains the *cat* gene flanked by *loxP* sites. (Rudner Lab collection)

pYY46 [*pMiniMAD P_{veg}-mCherry 395/396::P_{spoVA2}-spoVANJ2 kan (amp, erm)*] was constructed in a 3-way isothermal assembly reaction with two PCR products and pBa5 cut withy SpeI. The two PCR products were (1) the *spoVA2* promoter region and (2) the *spoVANJ2* gene, amplified from BaR1 genomic DNA using oYY196+oYY197 and oYY198+oYY199.

pYY48 [*ycgO::PsspB-optRBS-spoVA2(Bc) (ΔspoVANJ2) kan (amp)*] was constructed in a 3-way isothermal assembly reaction with two PCR products and pCB041 cut with EcoRI and XhoI. The regions upstream and downstream of *spoVANJ2* were amplified from pYG56 using oYY204+oYY205 and oYY203+oYY206. The two fragments were then inserted into the pCB041 by Gibson assembly. The resulting plasmid contains the *spoVA2(Bc)* operon with an in-frame deletion of *spoVANJ2*.

pYY49 [pMiniMAD Δ*dpaAB* (*amp*, *spec*)] was constructed in a 3-way ligation with two PCR products and pMiniMAD3 cut with BamHI and HindIII. The two PCR products were the regions upstream and downstream of the *dpaAB* operon*,* amplified from BaR1 genomic DNA using oYY209+oYY210 and oYY211+oYY212.

pYY50 [pMiniMAD Δ*dpaAB::kan* (*amp*, *spec*)] was constructed in a 2-way isothermal assembly reaction with a PCR product and pYY49 cut with SalI. The PCR product contained a *kan* cassette amplified from pWX470 using oYY213 and oYY214. pWX470 contains the *kan* gene flanked by *loxP* sites. (Rudner Lab collection)

pYY51 [pMiniMAD Δ*spoVAD1-his6* (*amp*, *spec*)] was constructed in a 3-way isothermal assembly reaction with two PCR products and pMiniMAD3 cut with SalI. The two PCR products were centered on the 3' end of the *spoVAD1* gene amplified from BaR1 genomic DNA using oYY216+oYY217 and oYY218+oYY219. The ligation of the PCR products generated an in-frame fusion of His6 to the C-terminus of SpoVAD1.

pYY52 [pMiniMAD Δ*spoVAD2-his6* (*amp*, *spec*)] was constructed in a 3-way isothermal assembly reaction with two PCR products and pMiniMAD3 cut with SalI. The two PCR products were centered on the 3' end of the *spoVA21* gene amplified from BaR1 genomic DNA using oYY220+oYY221 and oYY222+oYY223. The ligation of the PCR products generated an in-frame fusion of His6 to the C-terminus of SpoVAD2.

pYY54 [pMiniMAD Δ*spoVAEb2*(S65N) (*amp*, *spec*)] was constructed in a 3-way isothermal assembly reaction with two PCR products and pMiniMAD3 cut with SalI. The two PCR products were centered S65 in *spoVAEb2* and were amplified from BaR1 genomic DNA using oYY234+oYY235 and oYY236+oYY237. The ligation of the PCR products generated the S65N mutation.

pYY55 [*ycgO::PsspB-optRBS-spoVA2(Bc) (spoVAEb2-S65N) kan (amp)*] was constructed in a 3-way isothermal assembly reaction with two PCR products and pCB041 cut with EcoRI and XhoI. The two PCR fragments were amplified from pYG56 using oYY238+oYY239 and oYY240+oYY241. The resulting plasmid contains the *spoVA(Bc)* operon with S65N in the *spoVAEb2* gene.

pYY56 [*ycgO::PsspB-optRBS-spoVA2(B (ΔspoVANJ2 spoVAEb2-S65N) kan (amp)*] was constructed in a 3-way isothermal assembly reaction with two PCR products and pCB041 cut with EcoRI and XhoI. The two PCR fragments were amplified from pYY48 using oYY238+oYY239 and oYY240+oYY241. The resulting plasmid contains the *spoVA(Bc)* Δ*spoVANJ2* operon with S65N in the *spoVAEb2* gene.

pYY57 [pET-DUET *spoVAEb2(Bc)-proC His-SUMO-spoVAC2(Bc) (No FLAG) (amp)*] was constructed in a 3-way isothermal assembly reaction with two PCR products and pYG84 cut with SphI and XhoI. The two PCR fragments were amplified from pYG84 using oYY246+oYY247 and oYY248+oYY249. The resulting *E. coli* expression plasmid has His-SUMO-*spoVAC2* without a FLAG tag.

pYG56 [*ycgO::PsspB-optRBS-spoVA2(Bc) kan (amp)*] was constructed in a 3-way ligation with two PCR products and pCB041 cut with EcoRI and XhoI. One PCR product contains the PsspB promoter amplified with oYG117 and oYG118 using gDNA of *B. subtilis 168* as template, and cut with EcoRI and SpeI. The other PCR product contains *spoVA2(Bc)* amplified with oYG120 and oYG122 using gDNA of *B. cereus ATCC 14579*, and cut with SpeI and XhoI. pCB041 [*ycgO::kan* (*amp*)] is a *B. subtilis* double crossover integration vector at the *ycgO* locus with a *kan* cassette. (Rudner Lab stock)

pYG63 [*yhdG::PsspB-spoVA(Bs) spec (amp)*] was constructed in a 3-way ligation with two PCR products and pCB033 cut with EcoRI and XhoI. One PCR product contains the PsspB promoter amplified with oYG117 and oYG118 using gDNA of *B. subtilis 168* as template, and then cut with EcoRI and SpeI. The other PCR product contains *spoVA(Bs)* amplified with oYG148 and oYG149 using gDNA of *B. subtilis 168* as template, and then cut with SpeI and XhoI. pCB033 [*yhdG::spec* (*amp*)] is a *B. subtilis* double crossover integration vector at the *yhdG* locus with a *spec* cassette. (Rudner Lab stock)

pYG72 [*ycgO::PsspB-optRBS-spoVA2(Bc) (ΔspoVAC2) kan (amp)*] was constructed in a 3-way ligation with two PCR products and pCB041 cut with EcoRI and XhoI. One PCR product contains PsspB amplified with oYG117 and oYG118 using gDNA of *B. subtilis 168* as template, and then cut with EcoRI and SpeI. The other PCR product containing *spoVA2(Bc)* (Δ*spoVAC2*) was derived from overlap extension PCR of 2 fragments, one was amplified with oYG122 and oYG171 using gDNA of *B. cereus ATCC 14579,* and the second was amplified with oYG170 and oYG120 using gDNA of *B. cereus ATCC 14579.*

pYG73 [*ycgO::PsspB-optRBS-spoVA2(Bc) (ΔspoVAD2) kan (amp)*] was constructed in a 3-way ligation with two PCR products and pCB041 cut with EcoRI and XhoI. One PCR product containing P*sspB* was

amplified with oYG117 and oYG118 using gDNA of *B. subtilis 168* as template, and then cut with EcoRI and SpeI, and the other PCR product containing *spoVA2*(*Bc*)(Δ*spoVAD2*) was derived from overlap extension PCR of 2 fragments, one was amplified with oYG122 and oYG173 using gDNA of *B. cereus ATCC 14579,* and the second was amplified with oYG172 and oYG120 using gDNA of *B. cereus ATCC 14579.*

pYG74 [*ycgO::PsspB-optRBS-spoVA2(Bc)(ΔspoVAEb2) kan (amp)*] was constructed in a 3-way ligation with two PCR products and pCB041 cut with EcoRI and XhoI. One PCR product containing P*sspB* was amplified with oYG117 and oYG118 using gDNA of *B. subtilis 168* as template, and then cut with EcoRI and SpeI, and the other PCR product containing *spoVA2(Bc)*(Δ*spoVAEb2*) was derived from overlap extension PCR of 2 fragments, one was amplified with oYG122 and oYG175 using gDNA of *B. cereus ATCC 14579,* and the second amplified with oYG174 and oYG120 using gDNA of *B. cereus ATCC 14579.*

pYG84 [pET-DUET *spoVAEb2(Bc)-proC + His-SUMO-FLAG-spoVAC2(Bc) (amp)*] was constructed in 2-steps: (1) *spoVAEb2(Bc)* was amplified with primers oYG205 and oYG206 using gDNA of *B. cereus ATCC 14579* as template, and pLA73 (*MCS-proC His-SUMO-FLAG-MCS*) amplified with oYG190 and oYG191 to generate pYG84-1. (2) pYG84 was constructed in a 2-way isothermal assembly reaction with a PCR product containing *spoVAC2*(*Bc*) amplified with oYG207 and oYG208 using gDNA of *B. cereus ATCC 14579* as template, and pYG84-1 amplified with primers oYG192 and oYG193.

pYG85 [*pCOLADuet-spoVAD2(Bc)-His + spoVANJ2(Bc) (kan)*] was constructed in 2 steps: (1) *spoVAD2(Bc)* was amplified with primers oYG211 and oYG212 using gDNA of *B. cereus ATCC 14579* as template, and pCOLADuet-1 (Novagen) amplified with oYG200 and oYG167 to generate pYG85-1. (2) pYG85 was constructed in a 2-way isothermal assembly reaction with PCR product containing *spoVANJ2*(*Bc*) amplified with oYG209 and oYG210 using gDNA of *B. cereus ATCC 14579* as template, and pYG85-1 amplified with primers oYG198 and oYG199.

pYG702 [*pCOLADuet-spoVAD2(Bc)-His and lpp-retained-SS-spoVANJ2(Bc)-VSVG (kan)*] was constructed in a 2-way isothermal assembly reaction with a PCR product containing *lpp-retained-SS-spoVANJ2(Bc)-VSVG* and plasmid pYG85 amplified with primers oYG198 and oYG199. The PCR product was constructed in 3 steps: First, the *lpp-retained-SS-spoVANJ2-VSVG-1* was amplified with oYG1280 and oYG1288 using pYG85 as template. Second, and the *lpp-retained_SS-ylaJ-VSVG-2* was amplified with oYG770 and oYG1289 using *lpp-retained_SS-ylaJ-VSVG-1* as template. Third, *the lpp-retained_SS-ylaJ-VSVG* was amplified with oYG1287 and oYG1281 using *lpp-retained_SS-ylaJ-VSVG-2* as template. The resulting plasmid has a modified *spoVANJ2(Bc)* ORF that lacks its signal sequence and instead contains the signal peptide from *E. coli* Lpp. The Lpp signal sequence was further modified such that the lipidated protein is retained in the outer leaflet of the inner membrane. Finally the VSVG epitope tag was appended to the C-terminus of SpoVANJ2(Bc).

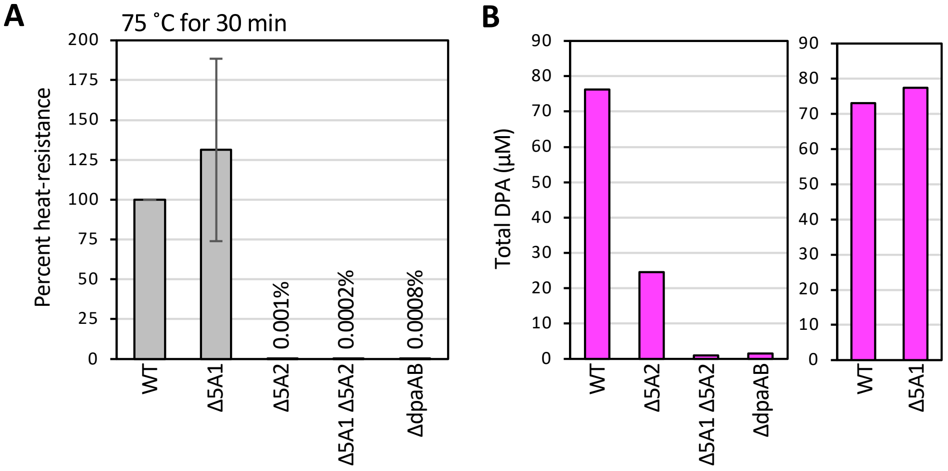

Figure S1. The *B. anthracis 5A2* locus is required for spore heat-resistance. (A) Bar graph of spore viability of the indicated strains after exposure to 75 °C for 30 min, as assayed by heat-resistant colony forming units (CFUs). Data are from three biological replicates (mean ± standard deviation). The viability of wild-type (WT) spores after 30 min at 75 °C was 37.4% of the spore viability after incubation at 65 °C for 30 min. **(B)** Bar graph showing total DPA in spores of the indicated strains. Spores were purified on a Histodenz step-gradient, normalized, and boiled for 30 min to release DPA. The supernatant was mixed with $TbCl_3$ and DPA:Tb^{3+} detected by fluorimetry. The two bar graphs are from separate experiments. Data shown are averages of three technical replicates.

A

strains	sporulation efficiency		
WT	100%	100%	100%
Δ5A1	89.3%	147.3%	110.1%
Δ5A2	8.4%	21.1%	20.4%
Δ5A1 Δ5A2	0.0010%	0.073%	0.00048%
ΔdpaAB	0.0008%	0.019%	0.0041%
D1-His	51.8%	114.9%	157.7%
D2-His	64.1%	122.3%	78.8%
D1-His Δ5A2	5.4%	23.8%	3.65%
D2-His Δ5A1	75%	94.7%	99.5%

B

strains	sporulation efficiency		
WT	100%	100%	100%
Δ5A1	95.6%	197.3%	100.8%
Δ5A2	<0.0002%	0.002%	0.001%
Δ5A1 Δ5A2	0.0006%	<0.0002%	<0.0002%
ΔdpaAB	0.0005%	0.002%	<0.0002%
D1-His	118.4%	281.3%	221.3%
D2-His	121.9%	509.8%	94.3%
D1-His Δ5A2	0.0009%	0.0006%	0.002%
D2-His Δ5A1	86.8%	159.4%	44.9%

C

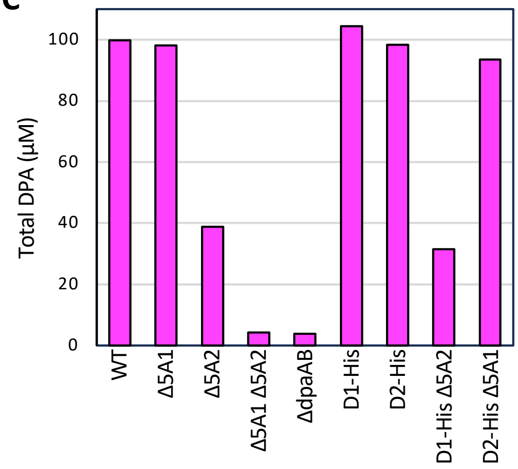

Figure S2. D1-His and D2-His are functional. (A) Table showing sporulation efficiencies of the indicated strains assessed by heat-resistant (65 °C for 30 min) colony forming units (CFUs). Three biological replicates are shown. **(B)** Table showing spore viability after exposure to 75 °C for 30 min, as assayed by heat-resistant CFUs. Three biological replicates are shown. The viability of wild-type (WT) spores after 30 min at 75 °C was 37.4% of the spore viability after incubation at 65 °C for 30 min. **(C)** Bar graph showing DPA levels in spores of the indicated strains. Spores were purified on a Histodenz step-gradient, normalized, and boiled for 30 min to release DPA. The supernatant was mixed with $TbCl_3$ and detected by fluorimetry. Representative data shown are averages of three technical replicates.

Figure S3
Yu et al.

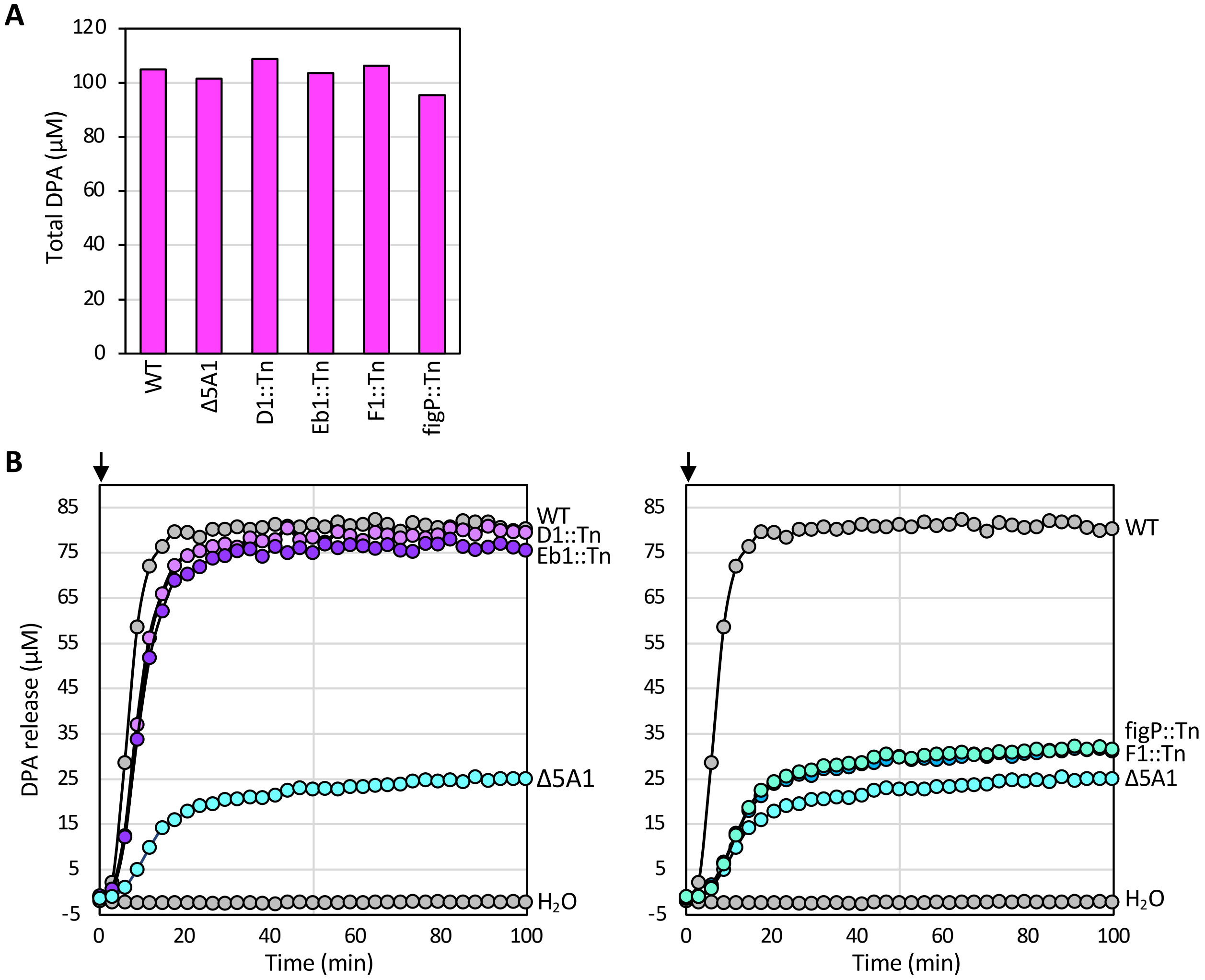

Figure S3. F1 and FigP are required for efficient germination. (A) Bar graph showing DPA levels in spores of the indicated strains. Spores were purified on a Histodenz step-gradient, normalized, and boiled for 30 min to release DPA. The supernatant was mixed with $TbCl_3$ and detected by fluorimetry. Representative data shown are averages of three technical replicates. **(B)** Germination assays using the spores from (A). Spores were incubated with 1 mM L-alanine and 1 mM inosine at time zero (arrows) and DPA release was monitored overtime at 30˚C using $TbCl_3$. Spores lacking D1 or Eb1 germinate at nearly wild-type rates due the presence of the *5A2* locus. Spores lacking FigP or F1 are impaired in germination and phenocopy Δ*5A1*. These data support the model that the *5A2* locus is sufficient for DPA release during germination and that *F1* is the only gene in the *5A1* locus that is critical for germination. These data are representative of three biological replicates.

Figure S4
Yu et al.

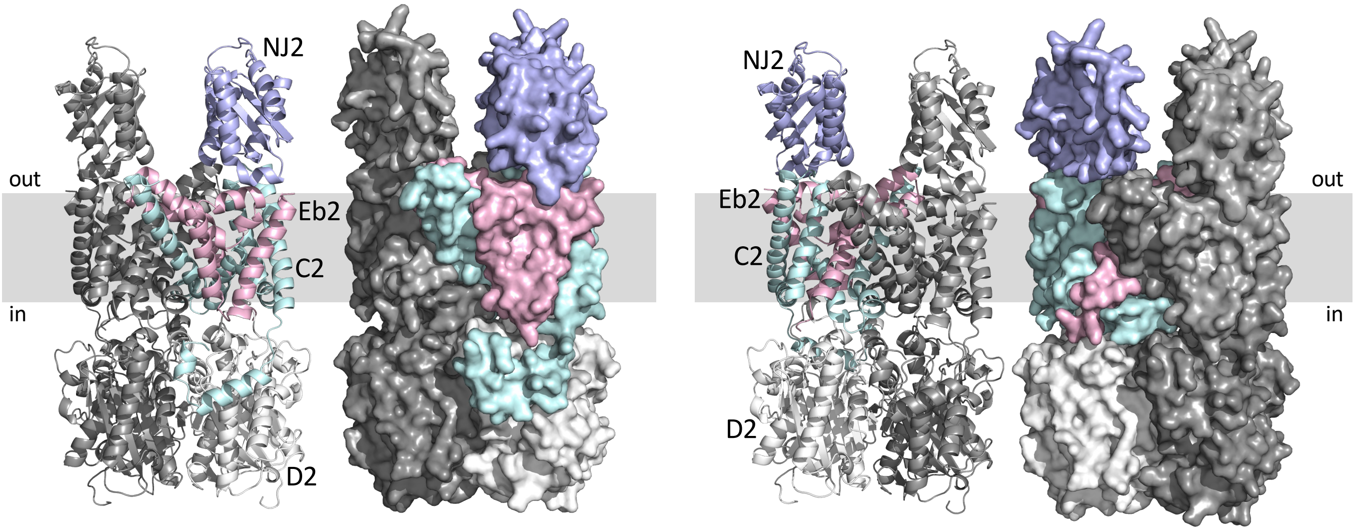

Figure S4. NJ2 is predicted to reside in a complex with C2, D2, Eb2. Side views of the AlphaFold3-predicted dimer of tetramers of the NJ2/C2/D2/Eb2 membrane complex. C2 (cyan) and Eb2 (pink) are predicted to form a membrane channel with interleaved transmembrane segments. D2 (white) is predicted to bind to the cytoplasmic face of the channel and interact with the N-terminus of C2 in the cytosol. NJ2 (light blue) is predicted to bind the extracytoplasmic face of the C2-Eb2 channel, resembling a "cap". The second tetramer is shown in grey. We note that AlphaFold3 predicts a low confidence heterodimer of 5A1 and 5A2 complexes.

sequence identity between *B. anthracis* and *B. cereus*	
NJ2	97%
C2	99%
D2	98%
Eb2	100%

Figure S5. Comparison of the proteins in the 5A2 DPA transport complex from *B. anthracis* and *B. cereus*. The four *B. anthracis* proteins that make up the 5A2 transport complex are ~98% identical to their *B. cereus* counterparts.

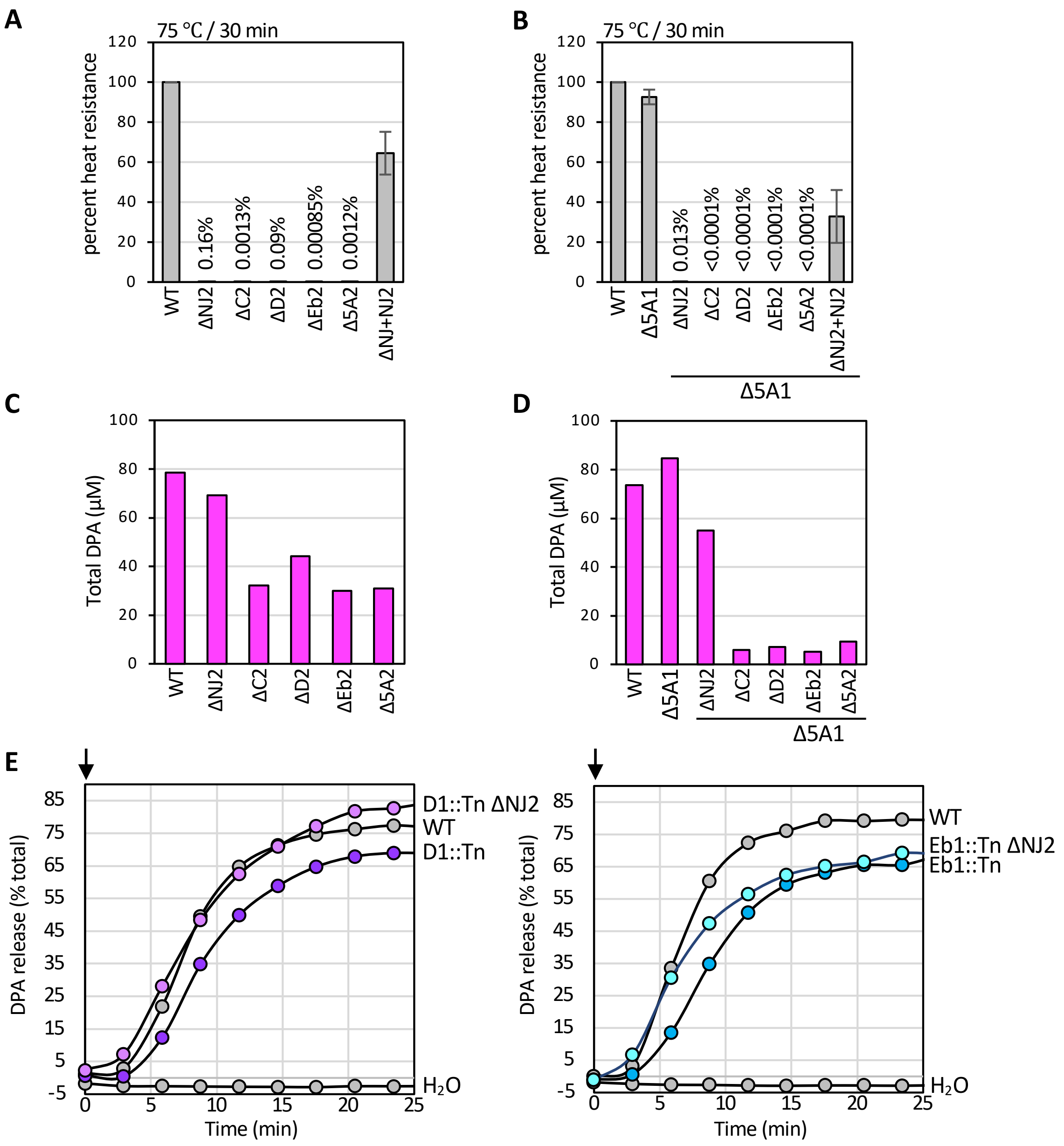

Figure S6. NJ2 is important for DPA import and impacts DPA export in *B. anthracis.* (A,B) Bar graphs of spore viability after exposure to 75 °C for 30 min, as assayed by heat-resistant colony forming units (CFUs). Data represent two biological replicates (mean ± standard deviation). Percentages are shown above the strains for which the spore viability was <1%. Viability of wild-type (WT) spores after 30 min at 75 °C was 37.4% of the spore viability after 30 min at 65 °C. **(C,D)** Representative bar graphs showing DPA levels in spores of the indicated strains. Spores were purified on Histodenz step-gradients, normalized, and boiled for 30 min to release DPA. The supernatant was mixed with $TbCl_3$ and detected by fluorimetry. Data shown are averages of three technical replicates. **(E)** Germination, as assayed by DPA release, of the indicated strains in response to 1mM L-alanine and 1mM inosine. Purified phase-bright spores were induced to germinate at time 0 (arrows) and the percentage of DPA release compared to the total spore DPA for each strain was plotted. WT spores incubated with water were used as a control. Spores lacking the a functional 5A1 transporter and *NJ2* reproducibly release DPA more rapidly than NJ2+ spores.

Figure S7
Yu et al.

A

ΔNJ2 suppressors

mutation	no. of times identified
Eb2(S65N)	3
gerE(AG)$_{-2}$	4
gerE(AG)$_{-1}$	5
gerE(AG)$_{+1}$	2

B

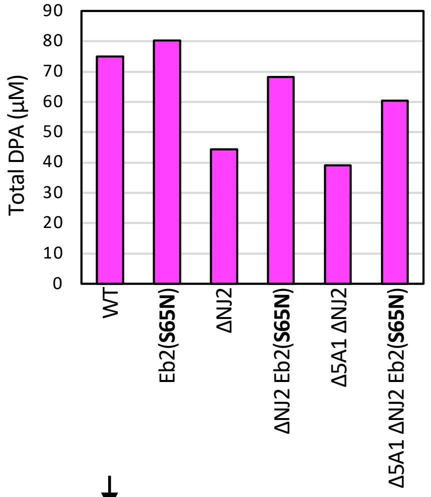

C

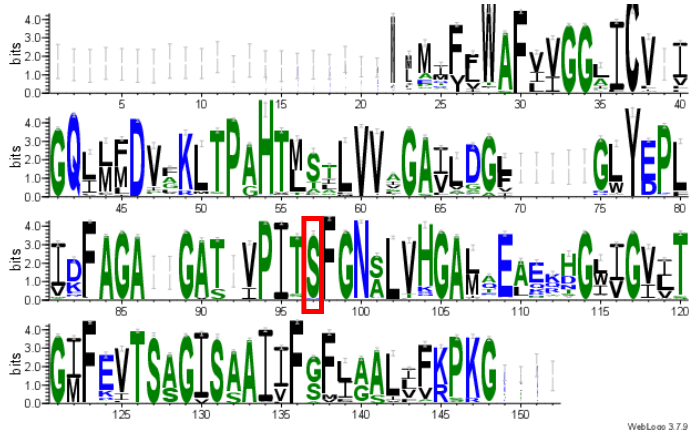

D

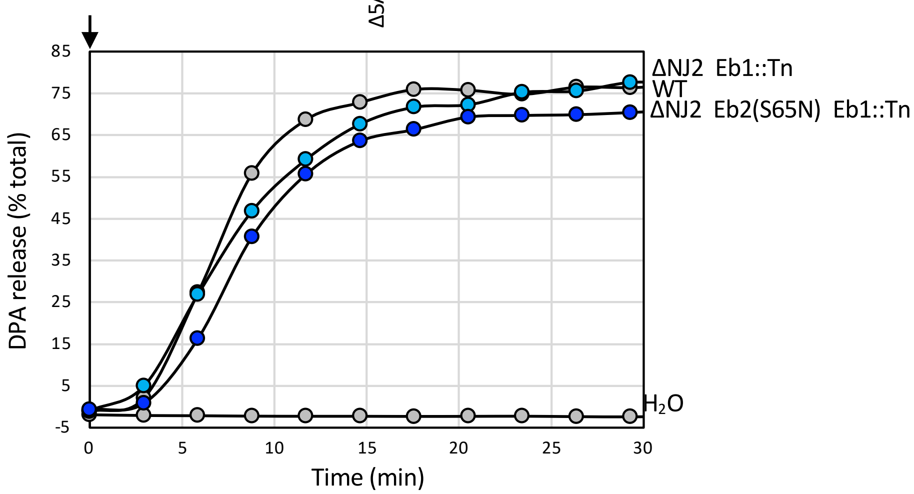

Figure S7. Eb2(S65N) bypasses the requirement for NJ2 in DPA import but slow DPA release during germination. (A) Table of all independently isolated suppressor mutations that restored heat resistance (75 ˚C for 30 min) to the ΔNJ2 mutant. Increase (+) or decrease (-) of the AG dinucleotide repeats at position 41 in the *gerE* open reading frame generates a frame-shift mutation. **(B)** Representative bar graph showing DPA levels in spores of the indicated strains. Spores were purified on a Histodenz step-gradient, normalized, and boiled for 30 min to release DPA. The supernatant was mixed with $TbCl_3$ and detected by fluorimetry. Data shown are averages of three technical replicates. **(C)** WebLogo highlighting conserved residues among Eb homologs. The *B. anthracis* Eb2 protein was used as a query in a PSI-BLAST search that was run through 5 iterations. The output file was aligned in Clustal Omega and visualized using the WebLogo3 Server. The serine residue that is equivalent to *B. anthracis* Eb2 S65 is boxed in red. Similar results were obtained when the *B. subtilis* Eb protein was used as the query. **(D)** Germination, as assayed by DPA release, of the indicated strains in response to 1mM L-alanine and 1mM inosine. Purified phase-bright spores were induced to germinate at time 0 (arrows) and the percentage of DPA release compared to total spore DPA for each strain was plotted. The Eb2(S65N) mutation slowed DPA release. WT spores were incubated with water as a control.

A

strains	sporulation efficiency
ΔgerA	81%
ΔgerA, Δ5A(*Bs*)	<0.000001%
ΔgerA, Δ5A(*Bs*), *5A2*(*Bs*)	62%
ΔgerA, Δ5A(*Bs*), *5A2*(*Bc*)	61%
ΔgerA, Δ5A(*Bs*), *5A2*(*Bc*) *ΔNJ2*	0.00002%
ΔgerA, Δ5A(*Bs*), *5A2*(*Bc*) *ΔC2*	<0.000001%
ΔgerA, Δ5A(*Bs*), *5A2*(*Bc*) *ΔD2*	<0.000001%
ΔgerA, Δ5A(*Bs*), *5A2*(*Bc*) *ΔEb2*	<0.00006%

B

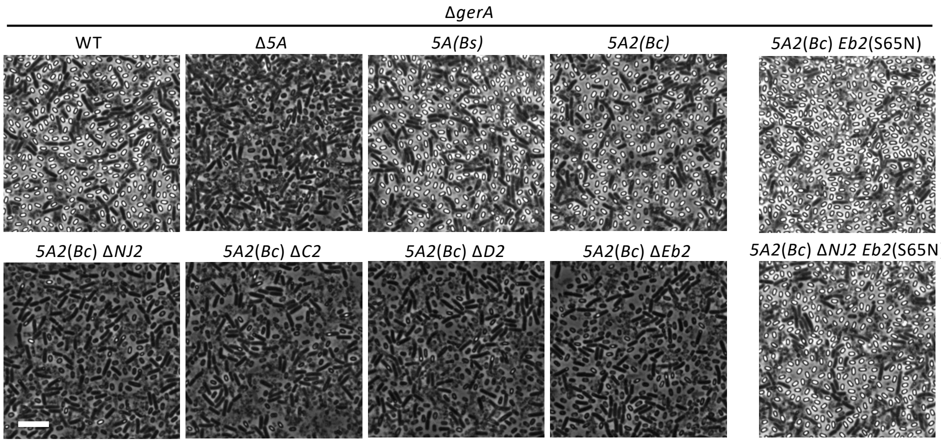

C

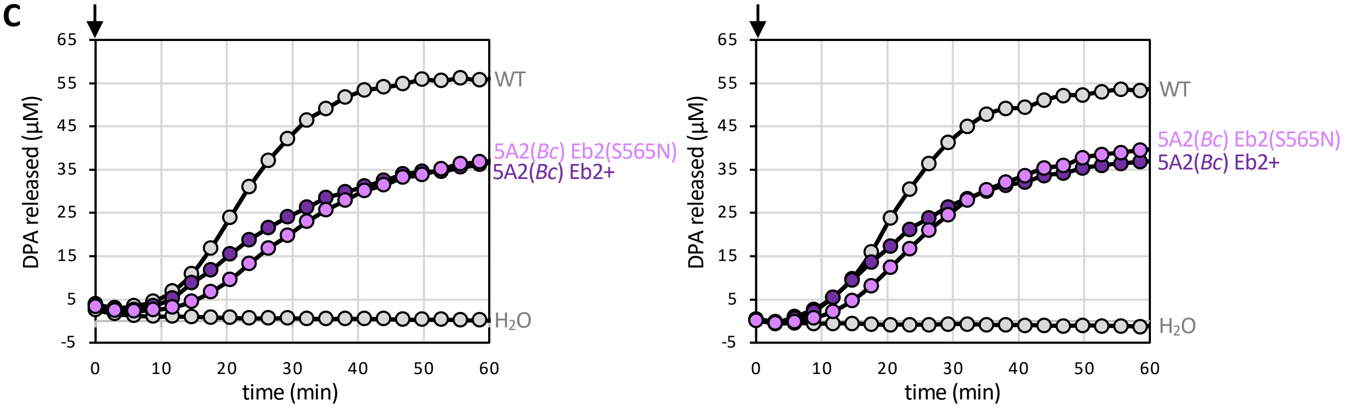

Figure S8. The NJ2 gene from *B. cereus* is required for sporulation in a *B. subtilis ΔgerA* mutant. (A) Table of sporulation efficiencies of the indicated *B. subtilis* strains as assayed by heat-resistant (80˚C for 20 min) colony forming units. Data represent two biological replicates (mean ± standard deviation). All strains lack the *gerA* locus to prevent GerA-mediated premature germination. **(B)** Representative phase-contrast images of sporulated cultures of the indicated strains. Scale bar, 5 µm. All strains lack the *gerA* locus. **(C)** Spore germination of the indicated strains that were *gerA+* in response to L-alanine as assayed by release of DPA. Spores were induced to germinate with 1mM L-alanine at time 0 (arrows), and DPA release was monitored over time. WT spores incubated with water were used as a control. The Eb2(S65N) spores released DPA more slowly than Eb2+. Two biological replicates are shown.

Figure S9
Yu et al.

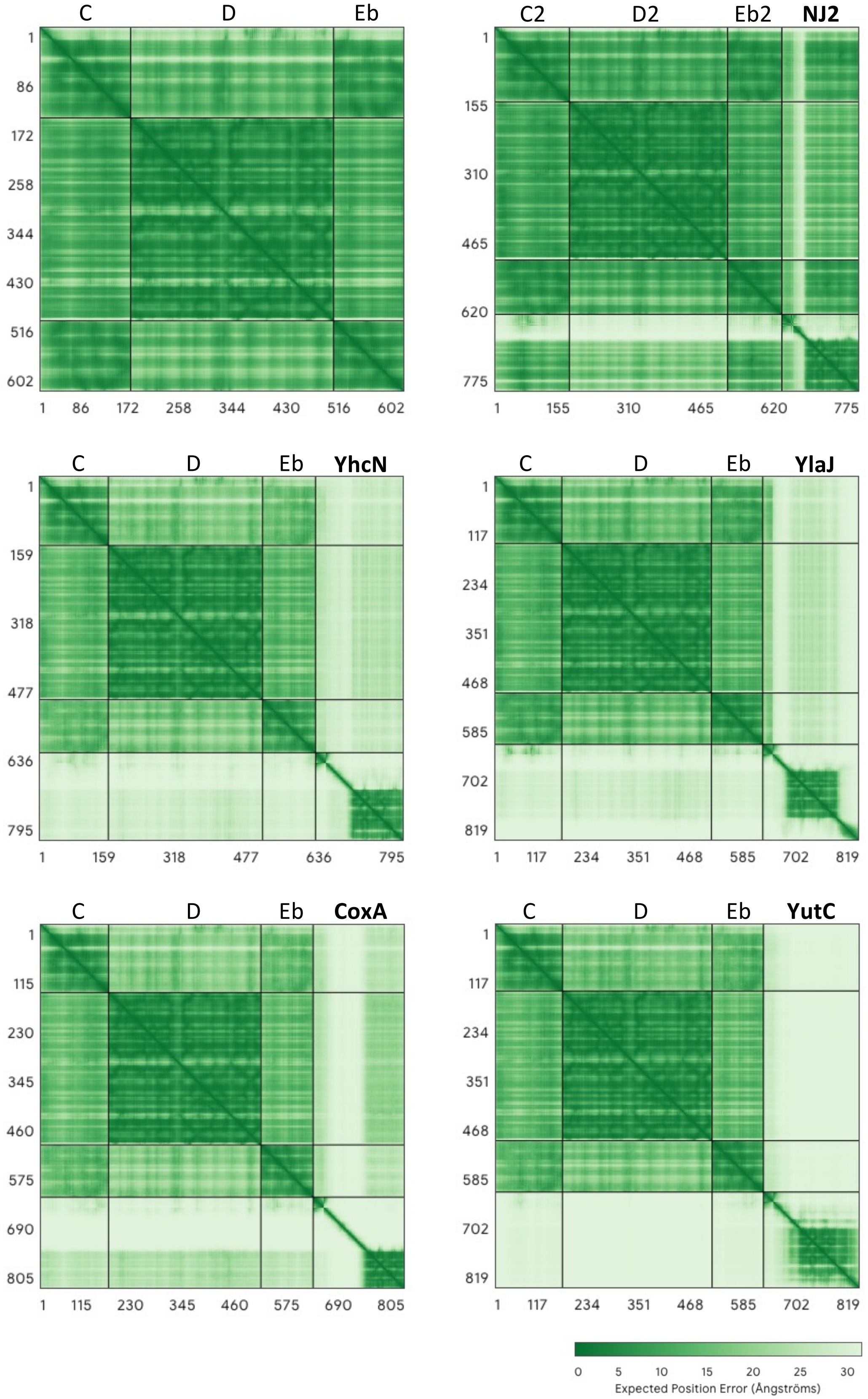

Figure S9 *B. subtilis* NJ2 paralogs are not predicted to interact with the the *B. subtilis* C/D/Eb transport complex. Predicted alignment error (pAE) plots from AlphaFold3-multimer predictions. *B. subtilis* C, D, Eb and *B. anthracis* C2, D2, Eb2, and NJ2 are predicted to form high-confidence complexes. The *B. subtilis* NJ2 paralogs YhcN, YlaJ, CoxA, and YutC are not predicted to interact with the *B. subtilis* C/D/Eb complex.

Table S1. *Bacillus* strains used in this study.

B. anthracis	**Genotype**	**Source**	**Figure(s)**
BaR1	wild-type *B. anthracis* (Sterne 9131)	James Kirby	1, 2, 4, 5, S1, S2, S3, S6, S7
BaR266	*spoVAD1::Tn spec*	This work	4E, S3AB, S6E
BaR267	*spoVAEb1::Tn spec*	This work	2H, 4E, S3AB, S6E
BaR269	*spoVAF1::Tn spec*	This work	2H, S3AB
BaR280	Δ*spoVAC2*	This work	4AB, S6AC
BaR287	Δ*spoVANJ2*	This work	4AB, 5AB, S6AC, S7B
BaR288	Δ*spoVAEb2*	This work	4AB, S6AC
BaR292	Δ*spoVAD2*	This work	4AB, S6AC
BaR298	Δ*spoVA2*	This work	2ABEFG, 4AB, S1AB, S2ABC, S6AC
BaR301	Δ*spoVA1*	This work	2ABEFGH, 4CD, S1AB, S2ABC, S3AB,S6BD
BaR330	Δ*spoVANJ2* Δ*spoVA1::cat*	This work	4CD, 5AB, S6BD, S7B
BaR331	Δ*spoVAC2* Δ*spoVA1::cat*	This work	4CD, S6BD
BaR332	Δ*spoVAD2* Δ*spoVA1::cat*	This work	4CD, S6BD
BaR333	Δ*spoVAEb2* Δ*spoVA1::cat*	This work	4CD, S6BD
BaR336	Δ*spoVA2* Δ*spoVA1::cat*	This work	2ABE, 4CD, S1AB, S2ABC, S6BD
BaR357	Δ*spoVANJ2 spoVAD1::Tn spec*	This work	4E, S6E
BaR363	Δ*spoVANJ2 spoVAEb1::Tn spec*	This work	4E, 5D, S6E, S7D
BaR377	Δ*spoVANJ2* Δ*spoVA1::cat 395/396*::P*spoVA2-spoVANJ2 kan*	This work	4C, S6B
BaR384	Δ*spoVANJ2 395/396::*P*spoVA2-spoVANJ2 kan*	This work	4A, S6A
BaR417	Δ*dpaAB::kan*	This work	2ABE, S1AB, S2ABC
BaR447	*spoVAD1-his6*	This work	2CD, S2ABC
BaR449	Δ*spoVA2 spoVAD1-his6*	This work	2D, S2ABC
BaR450	Δ*spoVA1 spoVAD2-his6*	This work	2D, S2ABC
BaR461	*spoVAD2-his6*	This work	2CD, S2ABC
BaR465	*spoVAEb2*(S65N)	This work	5AB, S7B
BaR466	Δ*spoVANJ2 spoVAEb2*(S65N)	This work	5AB, S7B
BaR468	Δ*spoVANJ2 spoVAEb2*(S65N) Δ*spoVA1::cat*	This work	5AB, S7B
BaR517	*figP::Tn spec*	This work	S3AB
BaR519	Δ*spoVANJ2 spoVAEb2*(S65N) *spoVAEb1::Tn spec*	This work	5D, S7D
B. subtilis	**Genotype**	**Source**	**Figure(s)**
BYY62	wild-type *Bacillus subtilis* 168 (trpC2)	Lab stock	6ABCD, S8C
BYY827	Δ*spoVA::tet* Δ*ycgO::erm*	This work	6AC
BYY828	Δ*spoVA::tet yhdG::PsspB-spoVA(Bs) spec*	This work	6ABCD
BYY829	Δ*spoVA::tet* Δ*ycgO::PsspB-optRBS-spoVA2(Bc) kan*	This work	6ABCD, S8C
BYY832	Δ*spoVA::tet* Δ*ycgO::PsspB-optRBS-spoVA2(Bc)* (Δ*spoVAC2*) *kan*	This work	6AC
BYY833	Δ*spoVA::tet* Δ*ycgO::PsspB-optRBS-spoVA2(Bc)* (Δ*spoVAD2*) *kan*	This work	6AC
BYY834	Δ*spoVA::tet* Δ*ycgO::PsspB-optRBS-spoVA2(Bc)* (Δ*spoVAEb2*) *kan*	This work	6AC
BYY838	Δ*spoVA::tet* Δ*ycgO::PsspB-optRBS-spoVA2(Bc)* (Δ*spoVANJ2*) *kan*	This work	6AC
BYY839	Δ*gerA::cat* Δ*spoVA::tet* Δ*ycgO::erm*	This work	S8AB
BYY840	Δ*gerA::cat* Δ*spoVA::tet*	This work	S8AB

	yhdG::PsspB-spoVA(Bs) spec		
BYY841	Δ*gerA::cat* Δ*spoVA::tet* Δ*ycgO::PsspB-optRBS-spoVA2(Bc) kan*	This work	S8AB
BYY843	Δ*gerA::cat* Δ*spoVA::tet* Δ*ycgO::PsspB-optRBS-spoVA2(Bc)* (Δ*spoVAC2) kan*	This work	S8AB
BYY844	Δ*gerA::cat* Δ*spoVA::tet* Δ*ycgO::PsspB-optRBS-spoVA2(Bc)* (Δ*spoVAD2) kan*	This work	S8AB
BYY845	Δ*gerA::cat* Δ*spoVA::tet* Δ*ycgO::PsspB-optRBS-spoVA2(Bc)* (Δ*spoVAEb2) kan*	This work	S8AB
BYY849	Δ*gerA::cat* Δ*spoVA::tet* Δ*ycgO::PsspB-optRBS-spoVA2(Bc)* (Δ*spoVANJ2) kan*	This work	6C, S8AB
BYY850	Δ*spoVA::tet* Δ*ycgO::PsspB-optRBS-spoVA2(spoVAEb2-S65N)(Bc) kan*	This work	6ABD, S8C
BYY851	Δ*spoVA::tet* Δ*ycgO::PsspB-optRBS-spoVA2(*Δ*spoVANJ2 spoVAEb2-S65N)(Bc) kan*	This work	6ABC
BYY852	Δ*gerA::cat* Δ*spoVA::tet* Δ*ycgO::PsspB-optRBS-spoVA2(spoVAEb2-S65N)(Bc) kan*	This work	S8B
BYY853	Δ*gerA::cat* Δ*spoVA::tet* Δ*ycgO::PsspB-optRBS-spoVA2(*Δ*spoVANJ2 spoVAEb2-S65N)(Bc) kan*	This work	S8B
BYY854	Δ*sleB::erm* Δ*gerA::cat*	This work	6B
BYY856	Δ*sleB::erm* Δ*gerA::cat* Δ*spoVA::tet* Δ*ycgO::PsspB-optRBS-spoVA2(Bc) kan*	This work	6B
BYY859	Δ*sleB::erm* Δ*gerA::cat* Δ*spoVA::tet* Δ*ycgO::PsspB-optRBS-spoVA2(Bc)* (Δ*spoVAD2) kan*	This work	6B
BYY864	Δ*sleB::erm* Δ*gerA::cat* Δ*spoVA::tet* Δ*ycgO::PsspB-optRBS-spoVA2(Bc)* (Δ*spoVANJ2) kan*	This work	6B
BYY866	Δ*sleB::erm* Δ*gerA::cat* Δ*spoVA::tet* Δ*ycgO::PsspB-optRBS-spoVA2(*Δ*NJ2 Eb2-S65N)(Bc) kan*	This work	6B
BYG793	Δ*gerA::cat*	This work	6C, S8AB

Table S2. Plasmids used in this study.

Plasmid	Description	Source
pETDuet-1	*T7 co-expression plasmid (amp)*	Novagen
pCOLADuet-1	*T7 co-expression plasmid (kan)*	Novagen
pAM174	*pBAD-ulp1 (cat)*	*Meeske et al.*
pMiniMAD	*Allelic exchange vector (amp, erm)*	*Patrick & Kearns*
pMiniMAD3	*Allelic exchange vector (amp, spec)*	*Mishra et al.*
pFR38	*Himar1C9 IR-spec (amp, erm)*	This work
pFR50	*pMiniMAD P_{veg}-mCherry (amp, erm)*	This work
pBaR5	*pMiniMAD P_{veg}-mCherry 395/396::kan (amp, erm)*	This work
pWX465	*loxP-cat-loxP (amp)*	Lab stock
pWX470	*loxP-kan-loxP (amp)*	Lab stock
pCB041	*ycgO::kan (amp)*	Lab stock
pYY31	*pMiniMAD3 ΔspoVANJ2 (amp, spec)*	This work
pYY32	*pMiniMAD3 ΔspoVAC2 (amp, spec)*	This work
pYY33	*pMiniMAD3 ΔspoVAD2 (amp, spec)*	This work
pYY35	*pMiniMAD3 ΔspoVAEb2 (amp, spec)*	This work
pYY36	*pMiniMAD3 ΔspoVA1 (amp, spec)*	This work
pYY37	*pMiniMAD3 ΔspoVA2 (amp, spec)*	This work
pYY40	*pMiniMAD3 ΔspoVA1::cat (amp, spec)*	This work
pYY46	*pMiniMAD P_{veg}-mCherry 395/396::P_{spoVA2}-spoVANJ2 kan (amp, erm)*	This work
pYY48	*ycgO::PsspB-optRBS-spoVA2(Bc) (ΔspoVANJ2) kan (amp)*	This work
pYY49	*pMiniMAD3 ΔdpaAB (amp, spec)*	This work
pYY50	*pMiniMAD3 ΔdpaAB::kan (amp, spec)*	This work
pYY51	*pMiniMAD3 ΔspoVAD1-his6 (amp, spec)*	This work
pYY52	*pMiniMAD3 ΔspoVAD2-his6 (amp, spec)*	This work
pYY54	*pMiniMAD3 ΔspoVAEb2(S65N) (amp, spec)*	This work
pYY55	*ycgO::PsspB-optRBS-spoVA2(Bc) (spoVAEb2-S65N) kan (amp)*	This work
pYY56	*ycgO::PsspB-optRBS-spoVA2(Bc) (ΔspoVANJ2 spoVAEb2-S65N) kan (amp)*	This work
pYY57	*pETDuet spoVAEb2-proC + His-SUMO-spoVAC2* (No FLAG*) (amp)*	This work
pYG56	*ycgO::PsspB-optRBS-spoVA2(Bc) kan (amp)*	This work
pYG63	*yhdG::PsspB-spoVA(Bs) spec (amp)*	This work
pYG72	*ycgO::PsspB-optRBS-spoVA2(Bc) (ΔspoVAC2) kan (amp)*	This work
pYG73	*ycgO::PsspB-optRBS-spoVA2 Bc (ΔspoVAD2) kan (amp)*	This work
pYG74	*ycgO::PsspB-optRBS-spoVA2 Bc (ΔspoVAEb2) kan (amp)*	This work
pYG84	*pETDuet spoVAEb2-proC + His-SUMO-FLAG-spoVAC2 (amp)*	This work
pYG85	*pCOLADuet spoVAD2-His + spoVANJ2 (kan)*	This work
pYG702	*pCOLADuet spoVAD2-His + lpp-retained-SS-spoVANJ2-VSVG (kan)*	This work

Patrick JE, Kearns DB. MinJ (YvjD) is a topological determinant of cell division in *Bacillus subtilis*. Mol Microbiol. 2008 70(5):1166-79.

A. Mishra et al. The SinR SlrR Heteromer Attenuates Transcription of a Long Operon of Flagellar Genes in *Bacillus subtilis* JMB 437 (2025) 169123

Meeske et al. SEDS proteins are a widespread family of bacterial cell wall polymerases. Nature. 2016 29;537(7622):634-638.

Table S3. List of oligonucleotide primers used in this study

primers	sequence
oFR295	GCCGGATCCATGATTTGGGATATTGGCGGA
oFR296	GCCGGGCCCCCGGTGTCATTATAATGATTG
oFR297	GCCGGGCCCCAGTTCATGTGACATGAACGT
oFR298	GCCGTCGACGGTAGACTACTACCTATCAAA
oYY147	GCCGAATTCGCGCAGCAAACACAAACGAT
oYY148	GCCGGATCCTTGTAACATTTATAGCCCTCCC
oYY150	GCCGTCGACCTTGCGGAAAGTGGTTCATC
oYY153	GCCGGATCCGTTTCGATTGAATTTGGAGGTG
oYY154	GCCGAATTCGTTAGGACTTTTTGACGCGC
oYY155	GCCGGATCCACTAGACATTGCGATCCTTCC
oYY156	GCCGGATCCAAAACGATTCTCGTTCAATGGG
oYY157	GCCGTCGACTGCCATTGTATGAGCTGGTG
oYY158	GCCGAATTCCATGCAGTGGTGCGGAAAGA
oYY159	GCCGGATCCGAGTTTTCGCATAATTATCATTC
oYY160	GCCGGATCCGAGTCTAACACTTAACGCTTTT
oYY161	GCCGTCGACCATAAGCAATTTCACGGCCG
oYY162	GCCGAATTCCACTGCCATATCAGGCACAA
oYY163	GCCGGATCCTACAGTCATTGAATGAAGCCC
oYY166	GCCGAATTCCGATCGGTTTGGTCAGTTTG
oYY167	GCCGGATCCAAAAATCATTGCGTTGCACCTC
oYY168	GCCGGATCCCCGAAAGGATAAGAGGTGTTC
oYY169	GCCGTCGACCTGCTTATGTATCTTCAACATC
oYY170	GCCGGATCCTTAAAAGAATAGATATCGAAAGAAC
oYY171	GCCGTCGACGAGCATAATGGGGATTATGAG
oYY172	GCCGAATTCGATAAGCCTGGACAGTTTGG
oYY173	GCCGGATCCTTGTTCCAATTCCATTCACCTC
oYY174	GCCGGATCCAGACAAAAATAAACGGGATTATAC
oYY175	GCCGTCGACTGGTGCAAACATCCCAGCTC
oYY190	GGTGAATGGAATTGGAACAAGGATCCTTCTGCTCCCTCGCTCAG
oYY191	AATCCCGTTTATTTTTGTCTGGATCCCAGGGAGCACTGGTCAAC
oYY196	CCGGTACCCATCCATGGGATACTAGTGTCGATAAATTATTATCAAGTAC
oYY197	ATGAGCCCGAGTTTTCGCATTGAATGAAGCCCCCTTTGGA
oYY198	TCCAAAGGGGGCTTCATTCAATGCGAAAACTCGGGCTCAT
oYY199	ATGGTTCGCTGGCTAGCCCTACTAGTTTAAGTGTTAGACTCCATTTTTTT
oYY203	GCGAAAACTCGAGTCTAACACTTAACGCTTTT
oYY204	GTGTTAGACTCGAGTTTTCGCATAATTATCATTC

oYY205	GATGTCACAAGCAGCTGGGA
oYY206	GCTGGGATCCATGCTAGCAT
oYY209	GCCGGATCCCCAAGAAGTACGTGAGCAAC
oYY210	GCCGTCGACAGTCAACATTCCCTAATTCACC
oYY211	GCCGTCGACTATATGAATTAAAAAACGAAAAAAAAC
oYY212	CGGAAGCTTACTGCAGTAGCTGGCATTGG
oYY213	AGGGAATGTTGACTGTCGACCAGGGAGCACTGGTCAAC
oYY214	TTTTAATTCATATAGTCGACTTCTGCTCCCTCGCTCAG
oYY216	GGGGATCCTCTAGAGTCGACGGAGGGGCTAGTATGAGGTT
oYY217	CACATGGTGATGATGGTGATGTTTCACTCTCTCAAATACGACACC
oYY218	ATCACCATCATCACCATGTGAAGGGAGAGTGAATTGTGGAT
oYY219	TTGCATGCCTGCAGGTCGACCGCCTAATCCAGCCGTTTGT
oYY220	GGGGATCCTCTAGAGTCGACCGATTCTCGTTCAATGGGGA
oYY221	ATGGTGATGATGGTGATGTCCACCAAATTCAATCGAAACGGCGTG
oYY222	GACATCACCATCATCACCATGGAGGTGCAACGCAATGATT
oYY223	TTGCATGCCTGCAGGTCGACTCTCCGCTCGGTTCTAAGAC
oYY234	GTACCCGGGGATCCTCTAGAGTCGACTCAATTTGTACTCGCAGGCG
oYY235	GTTACCGAAGTTTGTAATAGGTACGGTTGCCC
oYY236	CTATTACAAACTTCGGTAACGCACTCGTTC
oYY237	CCAAGCTTGCATGCCTGCAGGTCGACCAGAATGTAGCCAAGCTCGG
oYY238	GTCACAAGCAGCTGGGAAGG
oYY239	CATTACCAAAGTTTGTAATAGGTACAGTTGCCC
oYY240	CCTATTACAAACTTTGGTAATGCCCTCGTTC
oYY241	CTGGGATCCATGCTAGCATC
oYY246	CGCAAGGAATGGTGCATGC
oYY247	GGTTAAGTTTTTATCTTTACTAGCACCACCAATCTGTTCTCTGTG
oYY248	GCTAGTAAAGATAAAAACTTAACC
oYY249	GCAGCGGTTTCTTTACCAGA
oYG117	CAAGCGGAATTCGACTAGCTTAGCCTAAACGGCTAAG
oYG118	CTGGACTAGTTTTTTATTTAGTATGGTTGGGTTAACT
oYG120	CTGGCTCGAGCCCTGCTTATGTATCTTCAACATCATAAC
oYG122	CAGCGACTAGTACATAAGGAGGAACTACTATGACTGTAATTACGAAACTAAAGCAAAC
oYG148	GCGACTAGTAAGATGGTGATCAATGATGGAACGACGAATATTTATCCGGCT
oYG149	CTGGCTCGAGTTATGAATTGGTAGGCTGCCTTAAG
oYG167	CATATGTATATCTCCTTCTTATACTTAACTAATATACTAAGATGGGGAATTG
oYG170	TGGCTAGTAAACAATGGGGAGGGCTATAAATGTTACA
oYG171	CCTCCCCATTGTTTACTAGCCATTGCAATCCTTCCT
oYG172	GGCTATAAATGTTATCGATTGAATTTGGAGGTGCAACGCAATG
oYG173	CTCCAAATTCAATCGATAACATTTATAGCCCTCCCCATTGAAC

oYG174	CAATGATTCCGAAAGGATAAGAGGTGTTTATATGAC
oYG175	CACCTCTTATCCTTTCGGAATCATTGCGTTGCACCTCCA
oYG190	CATCCATGGTATATCTCCTTCTTAAAGTTAAAC
oYG191	GGAGGCTCTGGCGGAAGCGGAGGATCCA
oYG192	GGATCCTCCGCTTCCGCCGCTAGCT
oYG193	CTCGAGTCTGGTAAAGAAACCGCTGCTGCGA
oYG198	CATCCATGGTATATCTCCTTATTAAAGTTAAAC
oYG199	GGATCCGAATTCGAGCTCG
oYG200	CACCATCATCACCACCATCATCACTAGCTCGAGTCTG
oYG205	AGAAGGAGATATACCATGGATGATTTTTTTCTGGGCTTTCGTCA
oYG206	CCGCTTCCGCCAGAGCCTCCTCCTTTCGGTTTGAATAATAATGCTCCA
oYG207	AGCGGCGGAAGCGGAGGATCCGCTAGTAAAGATAAAAACTTAACCCCT
oYG208	GGTTTCTTTACCAGACTCGAGTTATAGCCCTCCCCATTGAACGAG
oYG209	CTTTAATAAGGAGATATACCATGGATGCAGTCATCCTTTTTCTATCAC
oYG210	CCGAGCTCGAATTCGGATCCTTAAGTGTTAGACTCCATTTTTTTACCA
oYG211	GTTAAGTATAAGAAGGAGATATACATATGTTACAAGGACACCGAACGTG
oYG212	TGATGGTGGTGATGATGGTGTTGCGTTGCACCTCCAAATTCAATC
oYG770	TTACTTTCCAAGTCGGTTCATCTCTATGTCTGTATAGGATCCTCCGCTTCCGCCA
oYG1280	TGTATAGGATCCTCCGCTTCCGCCAGAGCCTCCAGTGTTAGACTCCATTTTTTTACCA
oYG1281	GCCGAGCTCGAATTCGGATCCTTACTTTCCAAGTCGGTTCATCTCTATGTC
oYG1287	CTTTAATAAGGAGATATACCATGGATGAAAGCTACTAAACTGGTACTGGGCGCGGT
oYG1288	CTACTCTGCTGGCAGGTTGCGACCAGGGTTCGGATAATAGTCCTTTAGATAAAAAAGTG
oYG1289	CTAAACTGGTACTGGGCGCGGTAATCCTGGGTTCTACTCTGCTGGCAGGTTGCGACCA
